# An Early Examination: Psychological, Health, and Economic Correlates and Determinants of Social Distancing Amidst COVID-19

**DOI:** 10.3389/fpsyg.2021.589579

**Published:** 2021-08-11

**Authors:** Hohjin Im, Christopher Ahn, Peiyi Wang, Chuansheng Chen

**Affiliations:** ^1^Department of Psychological Science, University of California Irvine, Irvine, CA, United States; ^2^Department of Psychology, New York University, New York City, NY, United States

**Keywords:** health, socioeconomic, political, culture, social distancing, COVID-19, coronavirus

## Abstract

Federal and local government agencies were quick to issue orders for residents to shelter-in-place in response to the COVID-19 outbreak. This study utilized data collected from Unacast Inc., spanning observations of 3,142 counties across 50 states and the District of Columbia (*N* = 230,846) from March 8, 2020 to April 13, 2020 (*n* = 104,930) and from April 14, 2020 to May 24, 2020 (*n* = 131,912) in a 3-level multilevel model to examine the correlates of social distancing behavior, as measured by the relative reduction in (1) distance traveled and (2) non-essential visitations since baseline pre-COVID-19 times. Results showed that educational attainment and political partisanship were the most consistent correlates of social distancing. State-level indicators of culture appeared to have differentiated effects depending on whether the model outcomes were reduction in general mobility or to non-essential venues. State-level neuroticism was generally positively related to social distancing, but states marked by high neuroticism were slower to engage in such behaviors. Counties and states characterized as already engaging in preventive health measures (e.g., vaccination rates, preparedness for at-risk populations) enjoyed quicker engagement in social distancing. Specific implications of findings and future directions are discussed.

## Introduction

**Figure 1:**
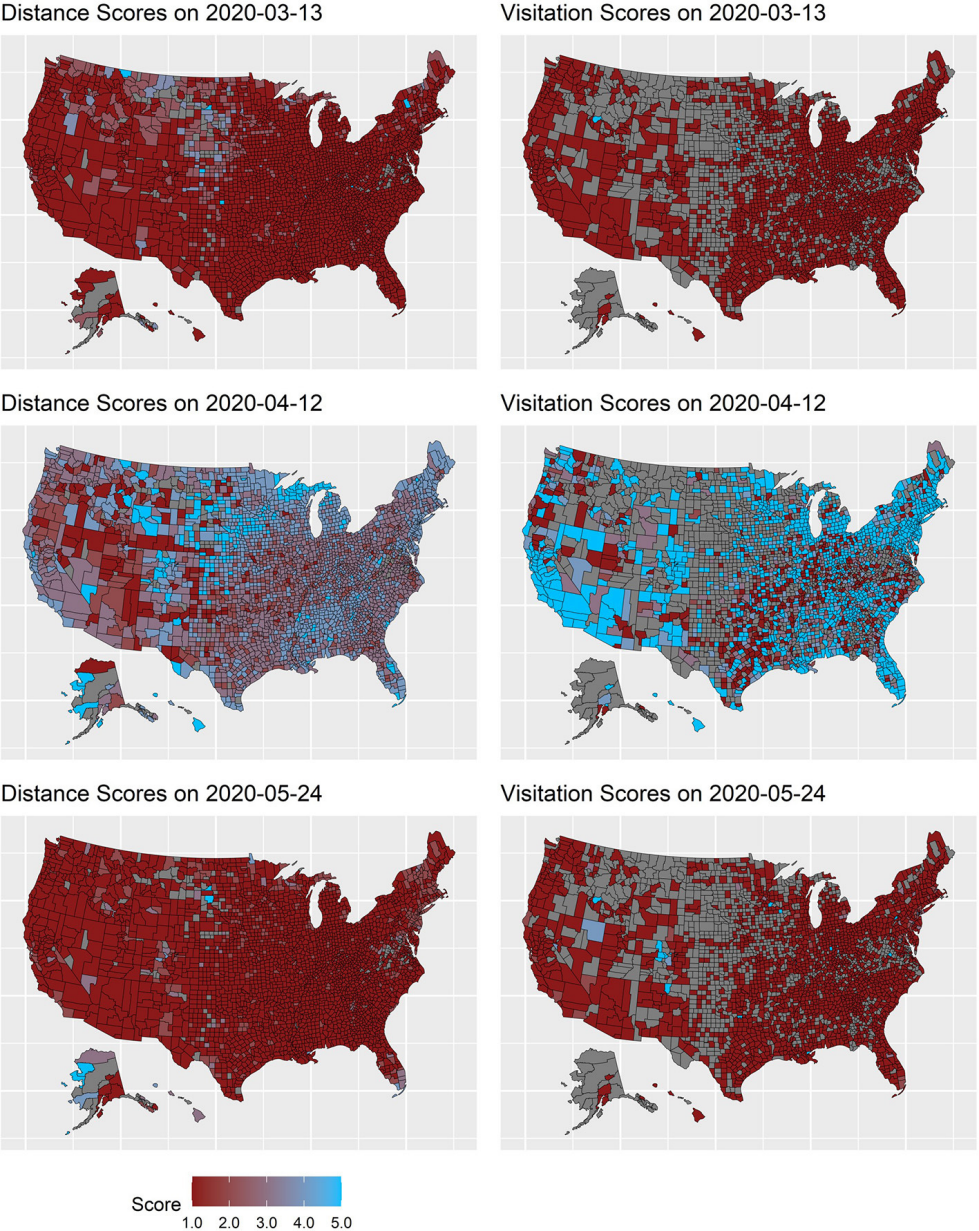
Distance and visitation scores across three timepoints. Numeric Grade scores from Unacast Inc. are shown for ease of visual interpretation. Scores correspond to the following: 1 = F, 2 = D, 3= C, 4 = B, and A = 5. Higher scores indicate greater social distancing. Distance scores were determined based on proportional reduction: A ≥ 70%, B = 55–70%, C = 40–55%, D = 25–40%, and F ≤ 25%. Visitation scores were based on: A ≥ 70%, B = 65–70%, C = 60–65%, D = 55–60%, and F ≤ 55%.

The advent of the COVID-19 pandemic has disrupted the lives of billions of people across the globe. Without a vaccine available for COVID-19 for the majority of 2020, many countries and local government agencies had implemented traditional public health interventions to curb viral spread, such as *social distancing*. In the United States, shelter-in-place orders had been issued throughout the nation in the early stages of the pandemic, mandating residents to limit their travel unless necessary (e.g., essential work or goods). Despite its limitations, social distancing was non-etheless endorsed by field experts to *flatten the curve* COVID-19 positive cases (Wilder-Smith and Freedman, 2020; Wilder-Smith et al., 2020). The United States collectively observed a peak reduction of ~55–70% in mobility during mid-April 2020 during the first wave of COVID-19 (Unacast, 2020). However, the country quickly regressed almost entirely to pre-COVID-19 trends in mobility within a few months (Unacast, 2020; Figure 1), possibly owing to social quarantine fatigue. Since the reopening of several states and districts in June, 2020, the majority of states have reported an uptick in positive COVID-19 cases, reaching new records for daily cases nationwide at the advent of its second wave (Johns Hopkins Coronavirus Resource Center, 2020).

### Political Partisanship and Social Distancing

News reports of segments of the population defying orders for social distancing had become all too ubiquitous entering the summer of 2020. Indeed, the threat of COVID-19 came at a peculiar time in human history, riddled with divisive political partisanship, widespread dissemination of misinformation, and radical extremism. Within the United States, Republican party leaders and their media pundits' alleged tendencies to downplay COVID-19 (e.g., Halon, 2020; Trump, 2020) have, at least on the surface, seem to have been adopted by their party contingents. Evidence in the early stages of the pandemic suggested that political conservatives reported less likelihood of engaging in preventive health behaviors (Everett et al., 2020; Im et al., 2021), less concern over COVID-19 (CIVIQS, 2020), and had greater tendency to believe in misinformation about COVID-19 (Gallup, 2020). Accordingly, recent studies show robust trends in which Republican partisanship and Conservative ideology were associated with less social distancing behaviors and intentions (Gollwitzer et al., 2020; Hsiehchen et al., 2020; Kavanagh et al., 2021; Kushner Gadarian et al., 2021). Indeed, as evidenced by prior studies, the overarching rhetoric and ideology endorsed by one's political party likely influenced its partisans' attitudes (Cohen, 2003) and denounced the efforts or initiatives from opposing parties (Levendusky, 2013). The political polarization raised a potential and prominent concern of creating partisan *echo chambers* whereby similar ideological values and preferences are recirculated amongst those who share them (Van Bavel and Pereira, 2018; Van Bavel et al., 2020). Given the observed trajectory and trends, the partisan divide is likely to continue playing a role in social distancing behavior. However, it remains unclear whether we may observe similar effects across different domains beyond political orientation. Understanding the correlates and determinants of social distancing may prove vital for appropriately addressing areas of further needed attention.

### Socioeconomic Status and Social Distancing

Decades of research into health behavior has illuminated various factors that may underlie one's motivation for engaging in preventive health behaviors. Prior reports suggest that those with higher and more formal education were more likely to utilize masks during times of influenza (Chuang et al., 2015), engage in prevention strategies for risky sexual behavior (Baker et al., 2011), and seek vaccination (Schwarzinger et al., 2010). In the case of Baker et al. (2011), formal education was one way in which one's higher order cognitive skills were developed, improving one's ability for health reasoning and utilization of preventative behaviors. Further, income had previously been documented to be positive correlates of public knowledge of H1N1 flu epidemic (Ho, 2012) while perceived financial barriers for appropriate response to H1N1 were related to concerns toward vaccination (Ford et al., 2009). Indeed, those on lower socioeconomic status (SES) steps may lack the financial resources that would otherwise allow them to stock up on essentials or take time away from essential work for strict self-isolation. On the other hand, individuals with higher SES may have been previously exposed to greater information about preventive health behaviors and be more likely to adhere to them. Thus, indicators of higher SES attainment may be positively related to engagement in social distancing behaviors and reduction of mobility.

### Social and Cultural Values and Social Distancing

It may also be important to consider personality or social values underlying preventive health behaviors. Prior research has found that individuals who score high on neuroticism, characterized by anxiety and sense of vulnerability (John and Sanjay, 1999), typically report higher than average levels of hypochondriacal symptoms (Noyes et al., 2003), express more anxiety over one's health or relevant viruses (Page et al., 2011; Boelen and Carleton, 2012), and desire more health testing (Johnson, 2000). Indeed, as neuroticism is acquainted by rumination and worry, it may be such that higher neuroticism was positively related to greater concern for COVID-19. This fear may ultimately translate to greater engagement in social distancing as a precautionary step to safeguard one's health.

Cultural tightness-looseness and collectivism may also play pivotal roles in the proper implementation of social distancing measures. Areas with high levels of tight cultures have stricter norms and are less tolerant of behavioral deviance whereas loose cultures have weaker norms and are more tolerant of deviant behaviors (Gelfand et al., 2011, 2017). When faced with threat, establishing strict social distancing norms may help to uphold and maintain adherence to social distancing measures and tighten communities together (Gelfand, 2020; Van Bavel et al., 2020). Indeed, recent studies have documented that tighter cultures observed better control of COVID-19 infection rates (Gelfand et al., 2020) and reducing mobility in response to COVID-19 outbreaks (Im and Chen, 2020). Thus, differences in cultural tightness across the United States may account for why some states performed much better than their counterparts in curbing the peak of the first COVID-19 wave.

Collectivism, the extent to which a cultural doctrine of interdependence and communal orientation is valued over individual needs (Triandis, 2001; Hofstede et al., 2010), may also be a key component in modeling social distancing behavior. In cultures where collectivism is endorsed, attention, and care for others is emphasized and one is compelled to adhere to their dutiful obligations for the sake of their neighbors (Hofstede et al., 2010). Early evidence of COVID-19 self-reported behavior lend credence to the notion of the importance of social obligations; in survey studies of adolescents, one's perceived social responsibility to others was positively related to engagement in preventative behaviors (Oosterhoff and Palmer, 2020; Oosterhoff et al., 2020). Further, recent studies have documented that those collectivism was related to greater intentions to reduce the spread of the virus (Biddlestone et al., 2020), don masks (Lu et al., 2021), and reduce mobility in response to COVID-19 (Im and Chen, 2020). Thus, cultural collectivism across the United States may also account for greater and prolonged reduction in mobility compared to their more individualistic counterparts.

### Health and Social Distancing

Lastly, measures of health, both at the state-level and county-level, may capture some motives underlying engagement in social distancing. At the macro-level, orders for behavioral change from government agencies may be effective but not sufficient. Indeed, Paton et al. (2008) recommended health agency representatives to act as consultants for communities to enact appropriate health behavior changes as opposed to directing needed changes. Such actions may allow better dissemination and communication of the health risks associated with COVID-19 at the local level, helping to facilitate the adoption of prevention-oriented behaviors. At the micro-level, individual perceptions may play larger roles; prior studies have illustrated that perceived susceptibility to relevant viruses increased intentions for hand washing (Miller et al., 2012) and likelihood of seeking vaccination (Yang, 2015). In other words, those who may be of poorer health may be more likely to engage in preventive health behaviors to protect themselves from possible infection (Champion and Skinner, 2008). This may be particularly important as people's perceptions of risk and risk-management behaviors are influenced by others (Lion et al., 2002). In communities where primary health care service may be poor, engagement in preventive health behaviors is likely to be integral as one's primary source of a “safety net” (i.e., primary hospital care) may be less than adequate. Thus, it may be such that communities and states that are better equipped to address the health needs of at-risk populations, disseminate epidemiological information to its residents, and more independent in managing their health will observe greater engagement in social distancing behavior.

### The Current Study and Analytical Plan

Although many studies have been conducted on COVID-19, and several recent studies have sought to examine the underlying motives for behavioral intentions (Biddlestone et al., 2020; Everett et al., 2020) and self-reported social distancing (Oosterhoff and Palmer, 2020; Oosterhoff et al., 2020), there is a dearth of research that comprehensively examines the numerous socioeconomic, psychological, cultural, and health determinants of social distancing. Further, utilizing a large-scale, nation-wide dataset allows for a representative analysis of social distancing behavior across a diverse set of demographics and to examine the robustness of prior findings of key correlates of preventative health behaviors at the macro-level. This study takes advantage of available large, archival datasets to merge variables together into aggregate units of analyses at the county and state level for secondary analyses. In doing so, we explore relevant variables across three domains: (1) socioeconomic/demographic variables, (2) psychological variables and cultural variables, and (3) health variables. Lastly, by utilizing longitudinal data, these sets of analyses examine both the tightening and loosening of social distancing across the nation amid the waning levels of social distancing across the country during the first wave of COVID-19 (Figure 1).

**Figure 2:**
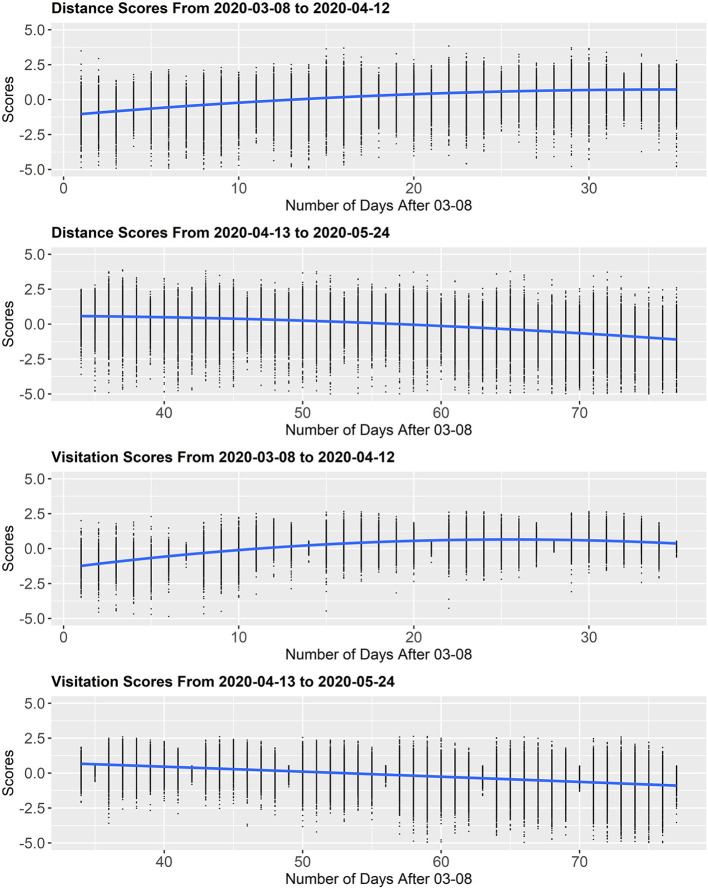
Mobility scores for distance and visitation from March 03 to May 24, 2020. All scores were logged and scaled by weekday; scores below −5 not shown in above graphs.

Using a total of 3,142 counties nested within 50 states and the District of Columbia, we utilized data available from February 24, 2020 to May 24, 2020 from Unacast Inc. in conjunction with publicly available data sources. Given the nature of the longitudinal and nested format of the available data (i.e., longitudinal measurements nested within counties nested within states), a three-level multilevel modeling analysis was used wherein Level 1 was each daily observation, Level 2 was the county, and Level 3 was the state. Further, because social distancing trends increased in the early stage of the US epidemic and have since declined, a segmented piece-wise multilevel modeling approach was used. The data were split into two components to capture (1) the incline in social distancing from March 08, 2020 (i.e., the first day termed *post-COVID-19* by Unacast Inc.) to April 12, 2020 where social distancing peaked for the country (Unacast, 2020) and (2) the subsequent decline in social distancing from April 13, 2020 to May 24, 2020 (Figure 2). Seven multilevel models tested the fixed and random effects of socioeconomic and demographic variables (Model 1), psychological and cultural variables (Model 3), and health variables (Model 5). Their interactions with progression of time (Models 2, 4, and 6) were also examined. A final, comprehensive model (7) tested the direct fixed and random effects of all relevant variables. To check that the significance of the interaction terms was not due to high collinearity with the other variables, interaction terms were all reexamined in isolation of other interaction terms. Results of these additional analyses can be found in the Supplementary Tables.

## Methods

### Sample

Spanning the 50 states (plus District of Columbia) and 3,142 counties, data were obtained through Unacast Inc. from February 24 to May 24, 2020 for a total sample of 272,818 daily observations at the county-level. Data from *pre-COVID-19* dates and immediately preceding the stock market crash (i.e., February 24 to March 07 as determined by Unacast Inc.) were removed from subsequent analyses, trimming the total sample examined in this study to 230,846 daily observations across the 3,142 counties. Data were split into two timeframes whereby the first timeframe from March 08 to April 12, 2020 marked the nationwide incline of social distancing (*n* = 104,930). The second timeframe from April 13 to May 24, 2020 marked the nationwide decline of social distancing (*n* = 131,912).

### Measures

Descriptive statistics for the time-invariant study variables at the county- and state-level are given in Table 1.

**Table 1:** Descriptive statistics for time-invariant variables.

	***N***	**Mean**	**95% Confidence interval**		**Standard deviation**
			**Lower**	**Higher**	
**County**					
Population density	2,998	261.89	197.13	326.64	1,808.92
County population	2,998	107,851.40	95,674.33	120,028.46	340,181.32
Older adult population	2,998	0.24	0.23	0.24	0.06
Education attainment	2,998	65.02	64.81	65.24	6.04
Income inequality	2,997	4.52	4.49	4.54	0.75
Personal income	2,998	4.40	4.35	4.44	1.25
Unemployment rate	2,998	4.14	4.09	4.19	1.44
Democrat proportion	2,982	0.32	0.31	0.32	0.15
Prevent hospitalization	2,988	4,884.66	4,818.73	4,950.60	1,838.82
Vaccination rate	2,991	41.89	41.54	42.23	9.58
Fair/Poor health rating	2,998	18.00	17.83	18.17	4.71
**State**					
Big 5 neuroticism	49	50.01	47.21	52.81	10.00
Cultural tightness	50	50.14	46.65	53.63	12.60
Cultural collectivism	50	50.08	46.94	53.22	11.34
Health community	51	3.12	2.88	3.36	0.88
Health epidemiology surv.	51	7.23	6.84	7.62	1.42

*Personal income in 10,000s; all variable statistics are given prior to standardization in subsequent analyses*.

#### Social Distancing (Mobility Scores)

Measures of social distancing were assessed by Unacast Inc. based on three key metrics: (1) the change in average distance traveled after March 9, 2020 (termed post-COVID-19 period), (2) the change in visitations to non-essential venues after COVID-19 period, and (3) absolute rate of possible unique human encounters made compared to pre-COVID-19 period. The third variable of human encounters was subsequently dropped upon post-analysis due to the high correlation with population density (e.g., *r* = 0.90+). Relative change in distance traveled served as a generic proxy measurement of mobility among the population to determine behavioral change after the start of the COVID-19 pandemic. Examining the change in *average distance traveled* allowed the scores to not be influenced by temporary or permanent changes in individuals' place of residency following the sudden advent of the pandemic that may have displaced certain subsets of the populations (e.g., students moving back home). Relative change to non-essential venues examined data pertaining to individuals' tendency to visit non-essential businesses (e.g., restaurants, hotels, etc.) compared to pre-COVID-19 times. The variables for change in distance and essential visitations were rated as percentile changes with values closer to −100(%) denoting greater engagement in social distancing *via* reduction in mobility. For ease of interpretation, all values were reverse coded so that higher values indicated greater social distancing.

Mobility scores were derived from geospatial mobility data provided to Unacast Inc. from app partners *via* collection from devices that offered location information. Data were aggregated at the respective population level (e.g., county, state) and no individual person, device, or household were identified from the data. Mobility scores were daily aggregated from tens of millions of devices offering geospatial data. For detailed methodology on the calculation and data sources, please refer to Unacast Inc. (https://www.unacast.com/post/the-unacast-social-distancing-scoreboard). Data from Unacast Inc. are not publicly accessible but requests for access may be directed to Unacast Inc.

#### Demographics

##### Population and Age

County total population data for 2019 were obtained through the U. S., Census Bureau (2020). Population density was calculated by dividing the state or county total population by their respective geographical area. Data measuring the proportion of older adults (ages 60+) from 2011 to 2015 for each county were obtained through the U. S. Census Bureau (2018).

##### Shelter-in-Place Order

State level shelter-in-place orders were dummy coded for each state starting at the date of the official announcement or date at which the order was due to take effect. For states where no state-level order was issued, county-level orders were used to dummy code at the county-level if available (Mervosh et al., 2020).

#### Socioeconomic Variables

##### Education Attainment

Data for educational attainment at the county level were weighted based on the proportion of adults with attainment of (1) less than high school diploma, (2) high school diploma, (3) some college, and (4) 4 years of college or higher, obtained through the U. S. Census Bureau (2019); U. S. Department of Agriculture (2020).

##### Personal Income

Average personal income at the county level for 2018 were obtained from the U. S. Bureau of Economic Analysis (2019).

##### Income Inequality

Income inequality was measured using the 2014–2018 statistics from the American Community Survey (ACS), provided by County Health Rankings (2020). Income inequality was determined by calculating the ratio of the 80th and 20th percentile for household income to denote the evident discrepancy between relative lower and upper SES class at each county.

##### Unemployment Rate

Unemployment rate was measured *via* the Bureau of Labor Statistics's 2018 data from the Local Area Unemployment Statistics (LAUS) program examining labor force data at different geographical levels of the country, provided by County Health Rankings (2020). Unemployment rate was calculated as the percentage of individuals at the county-level aged 16 and older who were unemployed but active in the labor market. Because high unemployment rate may be indicative of low economic opportunities, county-level unemployment rate serves as a proxy for local economic opportunity.

#### Psychological and Cultural Variables

##### Political Orientation

Measures of political orientation at the county and state level were both determined based on the 2016 US Presidential Election (Townhall, 2016). The proportion of registered votes for Democratic Candidate was used for each county to determine the basis of Democrat political orientation and degree of political engagement.

##### Neuroticism

State-level neuroticism data were obtained from a comprehensive cluster analysis of American individuals across 48 contiguous US states (*N* = 1,596,184) by Rentfrow et al. (2013) in measuring the Big 5 personality traits (John and Sanjay, 1999) of each US state. Big 5 personality scores for each state were determined *via* aggregating the mean of samples available across the 48 contiguous states.

##### Cultural Tightness-Looseness

State-level cultural tightness-looseness data were obtained from a composite aggregate of nine indicators of cultural tightness and looseness by Harrington and Gelfand (2014). Four indicators measured strength of punishment in schools and judicial system, two indicators measured permissiveness, two indicators measured moral order and behavioral constraint, and one indicator measured the proportion of foreign residents (Harrington and Gelfand, 2014).

##### Collectivism

State-level collectivism data were obtained from the composite aggregate of eight items: (1) percentage of single-residents, (2) percentage of elderly single-residents, (3) percentage of households with grandchildren residents, (4) divorce:marriage ratio, (5) percentage of non-religious residents, (6) percentage of Libertarian voters, (7) carpool:drive ratio among workers, and (8) percentage of self-employed workers. The items were standardized across states and established reliability and construct validity. All data were obtained from Vandello and Cohen (1999).

#### Health Variables

##### Preventable Hospitalization

Preventable hospitalization was measured using the 2017 data from the Centers for Medicare and Medicaid Services Office of Minority Health's Mapping Medicare Disparities (MMD) for hospitalization for 55 different medical conditions, as aggregated at the county-level by County Health Rankings (2020). Per County Health Rankings (2020), hospitalization for common outpatient treatable diagnoses is indicative of less than ideal quality of care in outpatient settings. Preventable hospitalization is thus meant to measure the quality of care and access to primary health care *via* the rate of hospitalization for outpatient typical diagnoses and conditions.

##### Vaccination

Vaccination against the seasonal flu was measured using the 2017 MMD data, as provided by County Health Rankings (2020). Per County Health Rankings (2020), vaccination rate was determined by the percentage of Medicare enrollees having received the annual flu vaccination within each county. Flu vaccination is a key preventive behavior and serves to be a proxy variable to measure the rate at which counties normally engage in preventive health behaviors.

##### Fair/Poor Health

Fair/Poor Health was measured using the 2017 BRFSS data, as aggregated and provided by County Health Rankings (2020). Per County Health Rankings (2020), the Fair/Poor Health variable was determined by the percentage of interviewed adults self-reporting poor or fair health condition in general. Because age is positively related to poorer health conditions, percentages reported were adjusted to account for different demographics in age categories.

##### Children and Other At-Risk Population

State-level preparedness in protecting at-risk individuals from pandemics and hazards (e.g., children, seniors, pregnant women) was aggregated *via* four indicators of specialized medical profession availability (2 indicators), proportion of youth with geographical access to trauma centers (1 indicators), and proportion of youth who missed school due to concerns over safety (1 indicator). All data were obtained from the National Health Security Preparedness Index (2019) as part of their Community Planning and Engagement Coordination Domain's subdomain (Children Other At Risk Populations).

##### Surveillance and Epidemiological Investigation

State-level surveillance of epidemiological threats (e.g., identification, discovery, monitoring, etc.) was aggregated *via* thirteen indicators measuring surveillance and reporting of threats (11 indicators) and availability of specialized medical and public health professionals (2 indicators). All data were obtained from the National Health Security Preparedness Index (2019) as part of their Health Security Surveillance Domain's subdomain (Health Surveillance Epidemiological Investigation).

## Results

### Tightening of Social Distancing for Distance Traveled

As expected, progression in time was the strongest predictor of greater reduction in mobility (β from 0.497 to 0.561, *p* all < 0.001) (Table 2). We also observed similar effects among counties with greater population (β from 0.057 to 0.063, *p* all < 0.001), education attainment (β from 0.102 to 0.141, *p* all < 0.001), personal income (β from 0.082 to 0.09, *p* all < 0.001), and unemployment rate (β from 0.056 to 0.078, *p* all < 0.001). Counties and states that issued shelter-in-place orders (β from 0.023 to 0.044, *p* all < 0.001) and have high proportion of elderly populations (β from 0.024 to 0.032, *p* from < 0.001 to 0.009) also observed greater reduction in mobility, albeit small effects. In Model 2, interactions between key SES variables and time indicated that the greater the income inequality (β = 0.034, 95% CI [0.030–0.039], *p* < 0.001), personal income (β = 0.013, 95% CI [0.008–0.019], *p* < 0.001), and education attainment (β = 0.042, 95% CI [0.036–0.048], *p* < 0.001) within the county, the quicker they were to reduce mobility. Model 3 examined the direct effects of psychological and cultural variables. Although counties with greater proportion of Democrats showed a slight positive effect of reduction in mobility (β = 0.024, 95% CI [0.002–0.047], *p* = 0.032), the relation between state neuroticism and greater reduction of mobility showed a much larger effect (β = 0.136, 95% CI [0.072–0.199], *p* < 0.001). In examining interactions (Model 4), counties with greater Democrat proportions (β = 0.062, 95% CI [0.058–0.066], *p* < 0.001) and states that were more collectivistic (β = 0.035, 95% CI [0.031–0.040], *p* < 0.001) and culturally tight (β = 0.03, 95% CI [0.026–0.035], *p* < 0.001) were quicker to reduce mobility. We observe a mitigating effect, albeit small, with states as they increase in neuroticism (β = −0.012, 95% CI [−0.015−0.008], *p* < 0.001). Model 5 examined the direct effects of health variables. Counties with poorer hospital quality showed greater reduction in mobility (β = 0.021, 95% CI [0.001–0.040], *p* = 0.036) whereas counties with greater proportion of unhealthy individuals showed the opposite effect (β = −0.038, 95% CI [−0.073−0.003], *p* = 0.035). However, both effect sizes were small. States that were better prepared to aid at-risk populations also reported a noticeable effect in reducing mobility (β = 0.086, 95% CI [0.034–0.138], *p* = 0.001). In examining interactions (Model 6), counties with more vaccination rates (β = 0.017, 95% CI [0.013–0.021], *p* < 0.001) and states better prepared to protect at-risk populations (β = 0.054, 95% CI [0.050–0.058], *p* < 0.001) were quicker to reduce mobility while counties with poorer hospital quality (β = −0.007, 95% CI [−0.011−0.003], *p* = 0.001) and states with better surveillance and epidemiological information unexpectedly observed a mitigation effect (β = −0.025, 95% CI [−0.028−0.021], *p* < 0.001).

**Table 2:** Reduction in distance mobility (From March 8 to April 12, 2020).

**Predictors**	**1**	**2**	**3**	**4**	**5**	**6**	**7**
	**β **	**β **	**β **	**β **	**β **	**β **	**β **
Intercept	0.01^***^	0.01^***^	−0.02^***^	−0.02^***^	0.01^***^	0.01^***^	−0.01^***^
Time	0.50^***^	0.50^***^	0.55^***^	0.55^***^	0.55^***^	0.56^***^	0.55^***^
Shelter-in-place order	0.04^***^	0.04^***^	0.04^***^	0.04^***^	0.04^***^	0.02^***^	0.04^***^
Population density	0.01	0.01	0.01	0.01	0.02	0.02	0.01
County population	0.06^***^	0.06^***^	0.06^***^	0.06^***^	0.06^***^	0.06^***^	0.06^***^
Older (60+) population	0.02^**^	0.02^**^	0.03^***^	0.03^***^	0.02^**^	0.02^**^	0.03^**^
Education attainment	0.14^***^	0.14^***^	0.14^***^	0.14^***^	0.13^***^	0.13^***^	0.10^***^
Income inequality	−0.01	−0.01^***^	−0.02^*^	−0.02^*^	−0.01	−0.01	−0.01
Personal income	0.08^***^	0.08^***^	0.09^***^	0.09^***^	0.09^***^	0.09^***^	0.08^***^
Unemployment	0.07^***^	0.07^***^	0.06^***^	0.06^***^	0.08^***^	0.08^***^	0.07^***^
Income inequality × Time		0.03^***^					
Personal income × Time		0.01^***^					
Education × Time		0.04^***^					
Democrat proportion			0.02^*^	0.02^***^			0.05^***^
Big5 neuroticism			0.14^***^	0.14^***^			0.11^***^
Tightness-looseness			−0.04	−0.04^*^			0.04
Collectivism			−0.01	−0.01^*^			−0.09^*^
Democrat × Time				0.06^***^			
Neuroticism × Time				−0.01^***^			
Tightness × Time				0.03^***^			
Collectivism × Time				0.04^***^			
Preventable hospitalization					0.02^*^	0.02^**^	0.02^*^
Vaccination					0.01	0.01	0.01
Fair/poor health					−0.04^*^	−0.04^*^	−0.09^***^
At-Risk preparedness index					0.09^**^	0.09	0.10^***^
Surveillance and epidemiological index					0.02	0.02	0.02
Hospitalization × Time						−0.01^***^	
Vaccination × Time						0.02^***^	
At-Risk × Time						0.05^***^	
Surveillance × Time						−0.02^***^	
**Random effects**							
σ2	0.50	0.50	0.32	0.31	0.32	0.32	0.32
τ00_county_	0.13	0.13	0.13	0.13	0.13	0.13	0.13
τ00_state_	0.07	0.07	0.04	0.04	0.05	0.05	0.03
ICC	0.28	0.29	0.36	0.36	0.36	0.36	0.33
N_county_	2997	2997	2977	2977	2987	2987	2973
N_state_	51	51	48	48	51	51	48
Observations	104895	104895	104195	104195	104545	104545	104055
Marginal R^2^/conditional R^2^	0.316/0.510	0.319/0.514	0.407/0.618	0.413/0.625	0.399/0.613	0.402/0.618	0.421/0.614

* *p < 0.05*;

** *p < 0.01*;

*** *p < 0.001*.

### Tightening of Social Distancing for Non-essential Venues

In examining the reduction in mobility to non-essential venues (Table 3), progression in time was again the largest predictor (β from 0.629 to 0.635, *p* from < 0.001 to 0.006). The issuance of shelter-in-place orders (β from −0.071 to −0.063, *p* all < 0.001) and counties with a higher proportion of older populations (β from −0.106 to −0.086, *p* all < 0.001) observed lower reduction in mobility to non-essential venues whereas counties with greater populations (β from 0.025 to 0.038, *p* from < 0.001 to 0.004), income inequality (β from 0.057 to 0.09, *p* all < 0.001), and educational attainment (β from 0.24 to 0.278, *p* all < 0.001), showed small to large effects in the opposite direction. Interactions (Model 2) indicate that counties with greater income inequality (β = 0.016, 95% CI [0.011–0.021], *p* < 0.001), personal income (β = 0.014, 95% CI [0.007–0.020], *p* < 0.001), and educational attainment (β = 0.035, 95% CI [0.028–0.042], *p* < 0.001) were quicker to reduce mobility to non-essential venues. Model 3 examined the direct effects of psychological and cultural variables on reducing non-essential mobility. Counties with greater proportion of Democrats (β = 0.075, 95% CI [0.054–0.097], *p* < 0.001) and states with higher neuroticism (β = 0.062, 95% CI [0.030–0.095], *p* < 0.001) observed greater reduction in mobility to non-essential venues whereas states with greater cultural tightness unexpectedly observed the opposite (β = −0.088, 95% CI [−0.127−0.050], *p* < 0.001). Interaction effects (Model 4) indicated that counties with greater proportion of Democrats (β = 0.04, 95% CI [0.035–0.046], *p* < 0.001) and states with greater collectivism (β = 0.013, 95% CI [0.008–0.018], *p* < 0.001) were quicker to reduce mobility to non-essential venues whereas states with greater neuroticism (β = −0.017, 95% CI [−0.022−0.012], *p* < 0.001) observed the opposite. Cultural tightness was not significant in Model 4 but was weakly significant in the isolated interaction model (see Supplemental Table E). Model 5 examined the direct effects of health variables on reducing non-essential mobility. Counties with greater proportion of vaccination (β = 0.067, 95% CI [0.047–0.086], *p* < 0.001) and fair/poor health (β = 0.108, 95% CI [0.072–0.143], *p* < 0.001) and states better equipped to aid at-risk populations (β = 0.057, 95% CI [0.023–0.090], *p* = 0.001) observed greater reduction of mobility to non-essential venues. On the other hand, counties with poorer hospital quality (β = −0.017, 95% CI [−0.022−0.012], *p* < 0.001) and states with greater surveillance of epidemiological issues (β = −0.006, 95% CI [−0.011−0.001], p = 0.013) were slower to reduce mobility to non-essential venues (Model 6).

**Table 3:** Reduction in non-essential visitation mobility (From March 8 to April 12, 2020).

**Predictors**	**1**	**2**	**3**	**4**	**5**	**6**	**7**
	**β**	**β**	**β**	**β**	**β**	**β**	**β**
Intercept	0.01^***^	0.01^***^	−0.01^***^	−0.02^***^	0.03^***^	0.03^***^	−0.01^***^
Time	0.63^***^	0.63^**^	0.63^***^	0.63^***^	0.63^***^	0.63^***^	0.63^***^
Shelter-in-place order	−0.06^***^	−0.06^***^	−0.06^***^	−0.07^***^	−0.06^***^	−0.07^***^	−0.06^***^
Population density	0.00	0.00	0.00	0.00	0.01	0.01	0.00
County population	0.04^***^	0.04^***^	0.03^**^	0.03^**^	0.04^***^	0.04^***^	0.03^**^
Older (60+) Population	−0.11^***^	−0.11^***^	−0.09^***^	−0.09^***^	−0.09^***^	−0.09^***^	−0.09^***^
Education attainment	0.26^***^	0.26^***^	0.24^***^	0.24^***^	0.28^***^	0.28^***^	0.25^***^
Income inequality	0.09^***^	0.09^***^	0.07^***^	0.07^***^	0.06^***^	0.06^***^	0.06^***^
Personal income	0.00	0.00	−0.01	−0.01	0.01	0.01	0.00
Unemployment	0.02	0.02	0.01	0.01	0.01	0.01	0.00
Income inequality × Time		0.02^***^					
Personal income × Time		0.01^***^					
Education × Time		0.04^***^					
Democrat proportion			0.08^***^	0.08			0.05^***^
Big5 neuroticism			0.06^***^	0.06^***^			0.05^**^
Tightness-looseness			−0.09^***^	−0.09^***^			−0.11^***^
Collectivism			0.02	0.02			0.01
Democrat × Time				0.04^***^			
Neuroticism × Time				−0.02^***^			
Tightness × Time				*0.00*			
Collectivism × Time				0.01^***^			
Preventable hospitalization					−0.02	−0.02	−0.01
Vaccination					0.07^***^	0.07^***^	0.07^***^
Fair/Poor health					0.11^***^	0.11^***^	0.08^***^
At-Risk preparedness index					0.06^***^	0.06	0.01
Surveillance and epidemiological index					0.00	0.00	−0.01
Hospitalization × Time						−0.02^***^	
Vaccination × Time						0.00	
At-Risk × Time						0.02^***^	
Surveillance × Time						−0.01^*^	
**Random effects**							
σ2	0.29	0.29	0.29	0.29	0.29	0.29	0.29
τ00_county_	0.07	0.07	0.07	0.07	0.06	0.06	0.06
τ00_state_	0.02	0.02	0.01	0.01	0.02	0.02	0.01
ICC	0.23	0.24	0.2	0.2	0.22	0.22	0.2
N_county_	2028	2028	2019	2019	2025	2025	2017
N_state_	51	51	48	48	51	51	48
Observations	70976	70976	70661	70661	70872	70872	70592
Marginal R^2^/Conditional R^2^	0.441/0.572	0.443/0.574	0.463/0.572	0.465/0.575	0.447/0.569	0.448/0.570	0.467/0.572

* *p < 0.05*;

** *p < 0.01*;

*** *p < 0.001; italicized interaction effects indicate non-robustness with single interaction models found in Supplementary Tables*.

### Loosening of Social Distancing for Distance Traveled

In examining the loosening of social distancing (Table 4), time was again the strongest predictor (β from −0.512 to −0.505, *p* all < 0.001). Counties with greater population (β from 0.069 to 0.094, *p* all < 0.001), population density (β from 0.014 to 0.022, *p* from 0.023 to 0.143), educational attainment (β from 0.098 to 0.2, *p* all < 0.001), personal income (β from 0.113 to 0.14, *p* from < 0.001 to 0.676), and unemployment (β from 0.031 to 0.089, *p* from < 0.001 to 0.01) resisted loosening of social distancing whereas counties with greater proportion of older populations observed the opposite (β from −0.086 to −0.047, *p* all < 0.001). The effect of population density was admittedly small and likely negligible whereas the effects of education attainment and personal income were the largest among SES variables. In examining the interactions (Model 2), counties with greater personal income levels were quicker to loosen social distancing (β = 0.033, 95% CI [0.029–0.037], *p* < 0.001). While the interaction between educational attainment and time was negative in Model 2 (β = −0.014, 95% CI [−0.018−0.009], *p* < 0.001), this result may have stemmed from collinearity as the effect was positive when examined in isolation (see Supplementary Table G). Model 3 examined the psychological and cultural variables related to the loosening of restrictions on mobility. Counties with higher proportion of Democrats (β = 0.16, 95% CI [0.137–0.184], *p* < 0.001) and states with greater neuroticism (β = 0.191, 95% CI [0.138–0.245], *p* < 0.001) were more persistent in limiting mobility to non-essential venues. In examining the interaction terms (Model 4), counties with greater proportion of Democrats (β = 0.024, 95% CI [0.021–0.028], *p* < 0.001) and states with greater neuroticism (β = 0.031, 95% CI [0.028–0.034], *p* < 0.001), tightness (β = 0.005, 95% CI [0.001–0.008], *p* = 0.021), and collectivism (β = 0.005, 95% CI [0.002–0.009], *p* = 0.005) were slower to loosen social distancing. Model 5 examined health variables in relation to loosening of social distancing. Counties with greater rates of vaccination (β = 0.026, 95% CI [0.005–0.047], *p* = 0.015) and proportion of fair/poor health (β = 0.078, 95% CI [0.039–0.117], *p* < 0.001) and states better equipped to aid at-risk populations (β = 0.111, 95% CI [0.038–0.185], p = 0.003) observed greater maintenance of social distancing. In examining interactions (Model 6), counties with greater proportion of poor hospital quality (β = 0.008, 95% CI [0.005–0.011], *p* < 0.001), vaccination rates (β = 0.026, 95% CI [0.022–0.029], *p* < 0.001), and states equipped to better aid at-risk populations (β = 0.012, 95% CI [0.009–0.016], *p* < 0.001) observed better maintenance of social distancing.

**Table 4:** Reduction in Distance Mobility (From April 13 to May 24, 2020).

**Predictors**	**1**	**2**	**3**	**4**	**5**	**6**	**7**
	**β **	**β **	**β **	**β **	**β **	**β **	**β **
Intercept	0.04	0.04^*^	−0.02^***^	−0.02	0.04^***^	0.04	−0.01^*^
Time	−0.51^***^	−0.51^***^	−0.51^***^	−0.51^***^	−0.51^***^	−0.51^***^	−0.51^***^
Shelter-in-place order	0.09	0.09	0.04	0.04	0.03	0.03	0.02
Population density	0.02^*^	0.02^*^	0.01	0.01	0.02^*^	0.02^*^	0.01
County population	0.09^***^	0.09^***^	0.07^***^	0.07^***^	0.09^***^	0.09^***^	0.07^***^
Older (60+) population	−0.09^***^	−0.09^***^	−0.05^***^	−0.05^***^	−0.07^***^	−0.07^***^	−0.05^***^
Education attainment	0.18^***^	0.18^***^	0.14^***^	0.14^***^	0.20^***^	0.20^***^	0.10^***^
Income inequality	0.01	0.01	−0.03^**^	−0.03^**^	−0.01	−0.01	−0.02
Personal income	0.14^***^	0.14	0.12^***^	0.12^***^	0.14^***^	0.14^***^	0.11^***^
Unemployment	0.09^***^	0.09^***^	0.03^*^	0.03^*^	0.07^***^	0.07^***^	0.04^***^
Income inequality × Time		0.00					
Personal income × Time		0.03^***^					
Education × Time		-0.01***					
Democrat proportion			0.16^***^	0.16^***^			0.19^***^
Big5 neuroticism			0.19^***^	0.19^*^			0.18^***^
Tightness-looseness			0.03	0.03			0.08^**^
Collectivism			−0.03	−0.03			−0.08^*^
Democrat × Time				0.02^***^			
Neuroticism × Time				0.03^***^			
Tightness × Time				0.00^*^			
Collectivism × Time				0.01^**^			
Preventable hospitalization					0.00	0.00^*^	0.01
Vaccination					0.03^*^	0.03^***^	0.02
Fair/poor health					0.08^***^	0.08^***^	−0.09^***^
At-Risk preparedness index					0.11^**^	0.11	0.07^*^
Surveillance and epidemiological index					0.01	0.01	0.03
Hospitalization × Time						0.01^***^	
Vaccination × Time						0.03^***^	
At-Risk × Time						0.01^***^	
Surveillance × Time						0.00*	
**Random effects**							
σ2	0.37	0.37	0.36	0.36	0.36	0.36	0.36
τ00_county_	0.19	0.19	0.18	0.18	0.19	0.19	0.18
τ00_state_	0.12	0.12	0.04	0.04	0.10	0.10	0.02
ICC	0.46	0.46	0.37	0.37	0.45	0.45	0.36
N_county_	2997	2997	2977	2977	2987	2987	2973
N_state_	51	51	48	48	51	51	48
Observations	131868	131868	130988	130988	131428	131428	130812
Marginal R^2^/conditional R^2^	0.353/0.650	0.354/0.651	0.416/0.634	0.418/0.636	0.372/0.654	0.373/0.655	0.426/0.632

* *p < 0.05*;

** *p < 0.01*;

*** *p < 0.001; italicized interaction effects indicate non-robustness with single interaction models found in Supplementary Tables*.

### Loosening of Social Distancing for Non-essential Venues

In examining the loosening of social distancing to non-essential venues (Table 5), time, as was the case for all preceding results, remained the strongest predictor of loosening mobility restrictions (β from −0.429 to −0.428, *p* all < 0.001). As was the case with loosening of general mobility restrictions, counties with greater proportion of older populations also observed a relatively strong negative effect (β from −0.165 to −0.123, *p* all < 0.001). On the contrary, counties with greater population (β from 0.027 to 0.054, *p* from < 0.001 to 0.012), income inequality (β from 0.057 to 0.116, *p* all < 0.001), and educational attainment (β from 0.228 to 0.314, *p* all < 0.001) saw greater maintenance of restricting mobility to non-essential venues. In examining the interaction terms (Model 2), counties with greater income inequality (β = 0.041, 95% CI [0.036–0.045], *p* < 0.001), personal income (β = 0.012, 95% CI [0.006–0.018], *p* < 0.001), and educational attainment (β = 0.041, 95% CI [0.035–0.047], *p* < 0.001) were also slower to loosen mobility restrictions. Model 3 examined the psychological variables in relation to the loosening of mobility to non-essential venues. While counties with higher proportion of Democrats were more persistent in limiting mobility to non-essential venues (β = 0.159, 95% CI [0.132–0.186], *p* < 0.001), cultural tightness had the opposite effect (β = −0.103, 95% CI [−0.161−0.046], *p* < 0.001). In examining interactions (Model 4), counties with greater proportion of Democrats (β = 0.064, 95% CI [0.059–0.069], *p* < 0.001) and states with greater collectivism (β = 0.033, 95% CI [0.028–0.038], *p* < 0.001) were slower to loosen mobility restrictions, while tighter states were quicker to do so (β = −0.034, 95% CI [−0.039−0.029], *p* < 0.001). Model 5 examined health variables. As was the case with the loosening of general mobility, counties with greater rate of vaccination (β = 0.076, 95% CI [0.052–0.100], *p* < 0.001) and fair/poor health (β = 0.184, 95% CI [0.139–0.229], *p* < 0.001) and states better equipped to aid at-risk populations (β = 0.075, 95% CI [0.027–0.124], *p* = 0.002) reported greater maintenance of restriction of mobility to non-essential venues. However, counties with greater proportion of poorer quality hospitals reported greater loosening of mobility (β = −0.028, 95% CI [−0.052−0.004], *p* = 0.023), albeit the small effect. In examining the interaction effects (Model 6), counties with poorer quality hospitals (β = −0.009, 95% CI [−0.013−0.004], *p* < 0.001) were quicker to loosen mobility restrictions whereas counties with greater rates of vaccination (β = 0.019, 95% CI [0.014–0.023], *p* < 0.001) and states better equipped to aid at-risk populations (β = 0.045, 95% CI [0.040–0.049], *p* < 0.001) observed the opposite effect.

**Table 5:** Reduction in essential visitation mobility (From April 13 to May 24, 2020).

**Predictors**	**1**	**2**	**3**	**4**	**5**	**6**	**7**
	**β **	**β **	**β **	**β **	**β **	**β **	**β **
Intercept	0.03^***^	0.03^***^	−0.00^**^	0.00	0.06^***^	0.06^***^	−0.00^***^
Time	−0.43^***^	−0.43^***^	−0.43^***^	−0.43^***^	−0.43^***^	−0.43^***^	−0.43^***^
Shelter-in-Place order	0.07^*^	0.07^*^	0.02	0.02	0.04	0.04	0.03
Population density	0.01	0.01	0.00	0.00	0.01	0.01	0.01
County population	0.05^***^	0.05^***^	0.03^*^	0.03^*^	0.05^***^	0.05^***^	0.03^**^
Older (60+) population	−0.16^***^	−0.16^***^	−0.13^***^	−0.13^***^	−0.14^***^	−0.14^***^	−0.12^***^
Education attainment	0.27^***^	0.27^***^	0.23^***^	0.23^***^	0.31^***^	0.31^***^	0.24^***^
Income inequality	0.12^***^	0.12^***^	0.07^***^	0.07^***^	0.07^***^	0.07^***^	0.06^***^
Personal income	0.00	−0.00^**^	−0.02	−0.02	0.01	0.01	−0.01
Unemployment	0.00	0.00	−0.03^*^	−0.03^*^	−0.02	−0.02	−0.03^*^
Income inequality × Time		0.04^***^					
Personal Income × Time		0.01^***^					
Education × Time		0.04^***^					
Democrat proportion			0.16^***^	0.16^***^			0.13^***^
Big5 neuroticism			0.03	0.03			0.02
Tightness-looseness			−0.10^***^	−0.10			−0.15^***^
Collectivism			0.07^*^	0.07^**^			0.08^*^
Democrat × Time				0.06^***^			
Neuroticism × Time				0.01*			
Tightness × Time				−0.03^***^			
Collectivism × Time				0.03^***^			
Preventable hospitalization					−0.03^*^	−0.03	−0.01
Vaccination					0.08^***^	0.08	0.07^***^
Fair/Poor health					0.18^***^	0.18^***^	0.10^***^
At-Risk preparedness index					0.08^**^	0.08^***^	−0.02
Surveillance and epidemiological index					−0.02	−0.02	−0.02
Hospitalization × Time						−0.01^***^	
Vaccination × Time						0.02^***^	
At-Risk × Time						0.04^***^	
Surveillance × Time						0.00	
**Random effects**							
σ2	0.54	0.54	0.54	0.53	0.54	0.54	0.54
τ00_county_	0.21	0.21	0.19	0.19	0.19	0.19	0.18
τ00_state_	0.06	0.06	0.03	0.03	0.06	0.06	0.03
ICC	0.33	0.33	0.29	0.29	0.31	0.32	0.28
N_county_	2028	2028	2019	2019	2025	2025	2017
N_state_	51	51	48	48	51	51	48
Observations	89140	89140	88744	88744	89023	89023	88671
Marginal R^2^/Conditional R^2^	0.330/0.552	0.333/0.556	0.371/0.553	0.378/0.560	0.339/0.546	0.342/0.549	0.376/0.551

* *p < 0.05*;

** *p < 0.01*;

*** *p < 0.001; italicized interaction effects indicate non-robustness with single interaction models found in Supplementary Tables*.

## Discussion

In our analyses of social distancing behaviors by US residents, our models examined the demographic, socioeconomic, psychological, cultural, and health correlates of social distancing across two key timeframes: (1) from March 08, 2020 (after the stock market crash) to April 12, 2020 (the peak of social distancing) and (2) from April 13, 2020 to May 24, 2020 to capture the decline of social distancing across the country during the first wave of the US COVID-19 outbreak. A summary of the direct effects of the study variables is given in Table 6.

**Table 6:** Summary of the size and direction of direct effects on social distancing mobility.

	**Incline of social distancing** **(March 8—April 12)**		**Decline of social distancing** **(April 13—May 24)**	
**Predictors**	**General mobility**	**Non-essential mobility**	**General mobility**	**Non-essential mobility**
**Demographic**				
Shelter-in-place order	0.04	−0.06	–	–
Population density	–	–	–	–
County population	0.06	0.03	0.07	0.03
Older (60+) population	0.03	−0.09	−0.05	−0.12
**Socioeconomic**				
Education attainment	0.10	0.25	0.10	0.24
Income inequality	–	0.06	–	0.06
Personal income	0.08	–	0.11	–
Unemployment	0.07	–	0.04	−0.03
**Psychological and cultural**				
Democrat proportion	0.05	0.05	0.19	0.13
Big5 neuroticism	0.11	0.05	0.18	–
Tightness-looseness	–	−0.11	0.08	−0.15
Collectivism	−0.09	–	−0.08	0.08
**Health**				
Preventable hospitalization	0.02	–	–	–
Vaccination	–	0.07	–	0.07
Fair/Poor health	−0.09	0.08	−0.09	0.10
At-Risk preparedness	0.10	–	0.07	–
Surveillance and epidemiological	–	–	–	–

*Color gradient from red (most negative) to green (most positive) respective to each model; non-significant coefficients in the comprehensive models (i.e., Model 7) not shown and marked as gray; all effects given as beta coefficients*.

### Demographic Variables

As expected, shelter-in-place orders put forth by counties and states served to reduce general mobility, albeit the effect was weak upon controlling for other variables. Surprisingly, the implementation of these orders had the opposite effect for social distancing for non-essential visitations. This presents a conundrum for policymakers as the restrictions to non-essential venues may result in some form of classical *reverse psychology* among residents. This may also stem from the fact that most states and counties merely implemented the orders but rarely legally enforced them (i.e., violators were not prosecuted). Future studies may examine whether residents abide by these orders more strictly upon observation of real consequences for violations. Further, there were no statistical differences between states that implemented shelter-in-place orders and those that did not for the latter end of the timeframe. This was also, unfortunately, limited in this analysis given that the reopening of various businesses and venues was much less homogenous than the closing. In other words, because many states and counties had their own policies for what businesses to open first, as well as the degree to which they may be open (e.g., mask policies, occupancy limits), it was impractical to binarily categorize open and closed conditions for states and counties with vastly qualitatively different policies. Future studies may opt to examine case studies of local or regional mobility statistics with respect to a more nuanced coding system of categorizing reopening policies to examine this relation more deeply.

County population remained a consistent and robust positive predictor of engaging in social distancing. That is, counties with greater population were more likely to reduce mobility as a whole and reduce travel to non-essential venues. However, the effect sizes for the latter were notably small and thus may have negligible practical outcomes in the long run. It is also worth noting that while absolute population count was a consistent predictor, population density yielded no significant correlation across all outcomes. This comes as a surprise given that one might conceptually reason that the risk of infectious spread is greater in densely populated areas and thus greater social distancing precautions are necessary to curtail this spread. Thus, even when absolute population counts are low, if population density itself is high, we would expect to see greater motivation to socially distance oneself from others and vice versa. However, the null results of population density in this study question that conjecture. Future research may seek to confirm these findings by examining how *perceptions* of density may or may not underlie motivation to engage in preventive health behaviors.

Lastly, results indicate that counties with greater proportion of individuals ages 60 and older were slightly better at social distancing for general mobility in the first half of the timeframe. However, this proportion was a robust and consistent negative predictor for social distancing for non-essential venues and both social distancing measures in the latter half of the timeframe. Given that COVID-19 has been deadlier for older age-ranges, we expected that counties with greater proportion of older populations to be more diligent in social distancing. However, the opposite trend was found. One explanation may be that because older populations also generally tend to be more conservative and traditional than their younger counterparts, they may be less receptive to adopting drastic changes to their livelihood. As will be discussed more in detail shortly, Republicans and culturally traditional (i.e., tight) tendencies among this population may account for such results.

### Socioeconomic Variables

Educational attainment was generally the most consistent and strong direct predictor of social distancing across all observed timepoints. On the other hand, income inequality was only a positive predictor of social distancing for social distancing in visitation to non-essential venues while personal income and income inequality were only significant for general reduction in mobility. Across all timeframes, educational attainment, income inequality, and personal income all served to generally quicken and prolong the maintenance of social distancing for both general mobility and visitation to non-essential venues. The results are in line with the proposition that the residents with greater personal income may have the financial resources to extensively socially distance oneself from the outside world, supporting general claims that income is a correlate of engagement in preventive health behaviors (Ford et al., 2009).

The degree of unemployment presents an interesting case. Given that high unemployment is typical of areas where a large portion of jobs may be unstable, it may be such that the sudden rise in unemployment in the late first and early second quarters have inadvertently forced many “blue-collar” job workers to stay home as businesses laid off workers or shuttered in response to government mandates. However, given that this variable predates the pandemic, the aforementioned conjecture warrants further study. This is particularly the case given the rapid changes residents made in response to the pandemic's impact on the labor market. Specifically, both income and educational attainment may provide some indication into how different SES classes may have been more negatively impacted by peripheral consequences of the pandemic. In this study, educational attainment remained the largest predictor of social distancing outside the progression of time. Although these results are consistent with prior findings (Schwarzinger et al., 2010; Baker et al., 2011; Chuang et al., 2015), the reasoning that these protective behaviors strictly stem from greater knowledge of diseases may fall short of adequately capturing the complex socioeconomic dynamic of the country during the early stages of the pandemic.

Indeed, individuals with higher educational attainment are more likely to have “white collar” jobs that may have been more viable to be transitioned to remote work whereas “blue collar” jobs, often manned by individuals on lower tiers of the SES ladder, typically do not share the same luxury. Further, the widespread unemployment during the early stages of the pandemic forced many households to seek flexible employment opportunities (e.g., gig economy), accelerating the employment sector's growth and economic standing (daVinci Payments, 2021). As lower SES individuals may have disproportionately entered the gig economy, the robust effects detected for educational attainment may partly reflect the changing labor market divide between socioeconomic classes.

### Psychological and Cultural Variables

Democrats generally tended to engage in more social distancing than their Republican counterparts and notably were better at prolonging the maintenance of mobility restrictions, serving as a buffer against the effect of time. This is at least in line with the anecdotal rhetoric across the country, where proponents of the Republican party seemed less receptive to engaging in preventive measures for COVID-19. Prior studies have also documented a harrowing divide between the two political parties whereby Democrats tended to be more receptive to the reliance of science in policy making (Blank and Shaw, 2015) and conservatives tended to be more skeptical toward scientific evidence incongruent with their prior attitudes and ideology (Kraft et al., 2015). Given the different rhetoric between political party leaders, at least early in the pandemic, it may have been that Democrats were more willing to trust that social distancing will yield promising results while Republicans were more distrusting of the motive underlying the movement for social distancing and/or other preventive health protocols. This not only complicates matters in that information must not only be communicated carefully but also be endorsed by partisan leaders who hold immense influence in tilting the attitudes of their partisan contingents (Cohen, 2003; Levendusky, 2013). Although future research is warranted to examine the nuances of the possible mechanisms underlying why Republicans may be more resistant to social distancing, our results were consistent with current evidence that partisanship remains a robust and consistent predictor of social distancing (Gollwitzer et al., 2020; Kushner Gadarian et al., 2021).

State-level neuroticism was a relatively consistent positive predictor of social distancing, in line with prior findings that neuroticism is related to greater concern for one's health (Noyes et al., 2003; Page et al., 2011; Boelen and Carleton, 2012). However, the peculiar set of findings was in how state-level neuroticism negatively interacted with time for both outcome variables during the incline of social distancing across the country. In other words, more neurotic states observed slower reduction in mobility. One proposition for this counter effect may be explained by the negative affective response individuals experience when socially pressured to abide by preventive health behavior norms (al Mamun et al., 2021), especially those exhibiting high neuroticism (Tucker et al., 2006). Indeed, because social distancing policies were largely implemented by state or local governments, these efforts may have been interpreted as these agencies exerting social control over its residents as opposed to encouraging positive social behavioral change. Thus, residents in states characterized by high neuroticism may have had negative responses to government instituted mandates for social distancing while still being motivated to engage in such behaviors to safeguard their own health, but simply preferring to do so at their own pace. Future research may examine how neuroticism personality traits may respond differently to preventive health behavior messages put forth by agencies of authority and how this response may either increase or decrease adherence to behavioral change.

What is most interesting, however, is the negative relation observed between collectivism and social distancing for general mobility. While prior studies would posit the opposite (Biddlestone et al., 2020; Im and Chen, 2020), we observe a negative trend in this data. However, we also see that, at least for the latter end of the current timeframe, that collectivism does indeed promote greater reduction to non-essential venues. Thus, this may suggest that collectivism may have differentiated effects on the types of social distancing. Similarly, we also observe that tightness-looseness follows a similar trend in which there is a robust negative effect of cultural tightness on the reduction of visitations to non-essential venues and an opposite effect for general mobility. Thus, tighter states observed less social distancing and were also quicker to loosen social distancing for non-essential visitations. Nonetheless, tighter states did observe greater social distancing for general mobility, at least consistent with global analyses of cultural tightness (Im and Chen, 2020). The results present a peculiar case; one proposition may be that cultural values appeal to target-specific preventive health behaviors and may warrant further nuancing in future studies.

### Health Variables

County-level rates of vaccination appeared to be relatively robust in being positively correlated with only social distancing for non-essential venues while state-level preparedness for aiding at-risk populations appeared to be robust for only social distancing for general mobility. Further, counties with higher rates of vaccination and states with better preparedness for at-risk populations also enjoyed quicker engagement in social distancing, supporting our prior propositions that populations already engaging in some forms of preventive health preparations ought to also combat the novel pandemic accordingly.

However, unexpectedly, counties with high rates of fair/poor health were consistently negatively correlated with social distancing for general mobility while positively correlated with social distancing for non-essential venues. While fair/poor health was originally hypothesized to result in a unidirectional effect of encouraging social distancing as a means to protect one's own health, the negative effect observed for general mobility suggests otherwise. Indeed, because having fair or poor status of health can be driven by a myriad of factors, the discrepancy in effects may likewise be driven by various underlying mechanisms not measured in this study. For instance, counties with high rates of deteriorating health may observe an uptick in mobility simply because those with poorer quality of health may already be engaging in risky health behaviors. On the other hand, we may observe greater social distancing for non-essential venues because residents in these counties may be lower on the SES ladder and do not have the financial leverage for leisure or travel. Due to the broad nature of the variable as a proxy, it is difficult to make conclusive statements regarding this variable and may be better suited, at least to the extent of this study, to be regarded as an interesting and peculiar control variable. Nonetheless, the results do seem to allude that one's perceived quality of health may yield interesting, possibly target-specific, effects for engagement in preventive health behaviors. Future research may assess this area of research.

County-level quality of hospitals and state-level surveillance and epidemiology monitoring capabilities yielded little to no effects across all models. This is somewhat surprising given that one might expect that poor quality of hospitals would remove the primary source of care one might have in the event of COVID-19 infection. Further, better state-level surveillance and epidemiology monitoring ought to suggest that states would enact quicker top-down responses to their local agencies to better inform residents of impending epidemic concerns or implement policies that aid in preventive behaviors. However, neither of these conjectures are supported in this study. One possibility may stem from the fact that both variables are proxies by nature and may not be accurate enough to capture the hypothesized phenomenon. Another possibility may be that both variables pose no real relation to social distancing. Indeed, given that social distancing is only one form of preventive health behaviors, these variables may in reality be correlated with other forms of preventive health behaviors (e.g., handwashing).

### Future Implications

Lastly, a point of interest and needed area of investigation involves examining what may be negative outcomes of social distancing and self-isolation. Although this area of inquiry goes beyond the scope of the current study, recent research has shown that social distancing can increase levels of depression and anxiety (Kämpfen et al., 2020; Marroquín et al., 2020) and exacerbate existing mental health issues (Murphy et al., 2020). Further, because stay-at-home orders and self-isolation can restrict physical activity, it remains important for individuals to remain vigilant in maintaining activities similar to pre-COVID-19 times (Jacob et al., 2020). Thus, while various psychological, economic, and health variables may promote social distancing, it may partly come at the cost of increasing susceptibility to other negative mental health outcomes should they be left to their own devices. Thus, future research examining the need for social interventions to promote mental health amid self-isolation may prove fruitful.

### Limitations

Several limitations should be considered in interpreting the results of the given study. Firstly, the use of physical mobility metrics as determined by GPS data offer objective measurements but do not allow for stringent accuracy in accounting for adherence to the 6 feet narrative pushed forth by state and federal US government agencies and all reports must be interpreted as proxies. Accordingly, this proxy-approach may not accurately reflect natural changes that occur from the changing economy or social ecology that the pandemic has brought nor can it accurately weight for different demographics who may opt into GPS tracking. Given this, interpretation of the results should be approached with caution, particularly in relation to several indicators of SES. Further, because several of the measures used in this study predate the pandemic, the extent to which the measures still provide accurate measures of regional differences should be inferred with caution, particularly for areas that were disproportionately affected by the pandemic. Future studies may benefit from supplementing subjective self-reports of social distancing with objective measures to more accurately model adherence to public directives.

Secondly, because widespread health directives pushed by government agencies are largely novel events within the US, the current model may not accurately map onto other countries, regions, or cultures where such directives may be more common. For instance, several East Asian countries have more recently dealt with other contagious viruses (e.g., MERS, SARS) or routinely face meteorological phenomena such as Yellow Dust that warrant routine government intervention. Future research may opt to examine social distancing behaviors across cultures to better understand how public policies may better address their denizens.

Thirdly, despite the temporal sequence of some variables, the lack of longitudinal data for predictor variables introduces standard limitations observed in cross-sectional studies and prohibits causal inferences. Accordingly, the current study is subject to cross-sectional dependence stemming from unobserved common factors at each time point. Given that most of the current study's research questions were contingent upon examining cross-level interactions spanning three units of analyses in comprehensive models, mixed effects modeling was used to implement both random and fixed effects. Nonetheless, future studies at a single level of analysis may opt to use second-generation unit root analyses to control for the unobserved common factors causing cross-sectional dependence more accurately.

## Conclusion

As the number of positive COVID-19 cases continue to grow every day, the need for implementation and execution of proper social distancing measures by the populace becomes ever more important. Because the efficacy of social distancing as a widescale traditional public health intervention is heavily contingent upon its residents doing one's own part, it is essential to monitor changes in foot traffic and travel to understand what factors underlie engagement in social distancing. While this study utilized data from the first wave of the US's COVID-19 outbreak, it provides an insight into the demographic, socioeconomic, psychological, cultural, and health correlates and determinants of social distancing in response to a novel virus. The variables measured in this study represent their underlying constructs as proxy variables and by no means are meant to be interpreted as inclusive of all variables relevant to the ongoing pandemic. Future studies are recommended to examine behaviors at the micro-level and confirm the robustness, or lack thereof, of the findings shown in this study.

As the US politics seemingly play a greater role than ever in the implementation of public health interventions and dissemination of information, it is important for federal and health agencies to be aware of how several factors may underlie responses to public health information. This is particularly considering recent documentation that political partisanship remains a consistent and robust correlate of attitudes toward science and the COVID-19 pandemic. Given the volatile trajectory of COVID-19 and human behavioral responses to it, the science community may benefit from continued research and input from social behavior scientists in uncovering motives for adhering to social distancing and other preventive health practices. Although the rapid dissemination of vaccines and normalization of masks continue in helping to contain the spread of COVID-19 within the US, the onset of several mutations and variants still pose imminent public health dangers. Examining the efficacy and importance of adherence to social distancing practices may prove critical in the near future.

## Data Availability Statement

The data analyzed in this study is subject to the following licenses/restrictions: Social distancing (mobility scores) measures are property of Unacast Inc and the authors are not at liberty to publicly disclose or provide access to the datasets. Request for access to the full mobility data may be directed to Unacast Inc. All other data sources are publicly available and may be accessed through the sources listed in the Methods section. Requests to access these datasets should be directed to Unacast Inc., info@unacast.com.

## Author Contributions

HI was responsible for compiling variables, creating a workable dataset, analyzing the data, and writing the manuscript. CA was responsible for aggregating relevant variables, data management, and drafting portions of the manuscript. PW was responsible for drafting and revising portions of the manuscript. CC was responsible for guiding the project from inception to completion in areas of writing, statistical analysis, and theory development. All authors contributed to the article and approved the submitted version.

## Conflict of Interest

The authors declare that the research was conducted in the absence of any commercial or financial relationships that could be construed as a potential conflict of interest.

## Publisher's Note

All claims expressed in this article are solely those of the authors and do not necessarily represent those of their affiliated organizations, or those of the publisher, the editors and the reviewers. Any product that may be evaluated in this article, or claim that may be made by its manufacturer, is not guaranteed or endorsed by the publisher.
