# Supplementary material for: An Early Examination: Psychological, Health, and Economic Correlates and Determinants of Social Distancing Amidst COVID-19
Source: Front Psychol. 2021 Aug 11;12:589579. doi: 10.3389/fpsyg.2021.589579 (PMC8385125; doi:10.3389/fpsyg.2021.589579)
Supplement: Supplementary file 1 [file Data_Sheet_1.docx]

***Supplementary Materials***

**1 Supplementary Tables**

Tables A-I provide the models given in Tables 1-4 in the main manuscript. Standardized 95% confidence intervals are given in parentheses below the beta coefficients. Interaction terms are run one at a time, serving as statistical robustness checks to Tables 1-4 to verify that the interaction terms were not due to collinearity.

- 1. **Change in Distance by Category of Variables for Time Frame 1**

Table A. Social Distancing (Reduction in Mobility) from March 08 to April 12 (SES)

|  | **1** | **2** | **3** | **4** |
| --- | --- | --- | --- | --- |
| **Predictors** | **β** | **β** | **β** | **β** |
| Intercept | 0.01 *** | 0.01 *** | 0.01 *** | 0.01 *** |
|  | (-0.06 – 0.09) | (-0.06 – 0.09) | (-0.06 – 0.09) | (-0.06 – 0.09) |
| Time | 0.50 *** | 0.50 *** | 0.50 *** | 0.50 |
|  | (0.49 – 0.50) | (0.49 – 0.50) | (0.49 – 0.50) | (0.49 – 0.50) |
| Shelter-in-Place Order | 0.04 *** | 0.04 *** | 0.04 *** | 0.04 *** |
|  | (0.03 – 0.05) | (0.03 – 0.05) | (0.03 – 0.05) | (0.03 – 0.05) |
| Population Density | 0.01 | 0.01 | 0.01 | 0.01 |
|  | (-0.00 – 0.03) | (-0.00 – 0.03) | (-0.00 – 0.03) | (-0.00 – 0.03) |
| County Population | 0.06 *** | 0.06 *** | 0.06 *** | 0.06 *** |
|  | (0.04 – 0.07) | (0.04 – 0.07) | (0.04 – 0.07) | (0.04 – 0.07) |
| Elder (60+) Proportion | 0.02 ** | 0.02 ** | 0.02 ** | 0.02 ** |
|  | (0.01 – 0.04) | (0.01 – 0.04) | (0.01 – 0.04) | (0.01 – 0.04) |
| Education Attainment | 0.14 *** | 0.14 *** | 0.14 *** | 0.14 *** |
|  | (0.12 – 0.16) | (0.12 – 0.16) | (0.12 – 0.16) | (0.12 – 0.16) |
| Income Inequality | -0.01 | -0.01 *** | -0.01 | -0.01 |
|  | (-0.03 – 0.01) | (-0.03 – 0.01) | (-0.03 – 0.01) | (-0.03 – 0.01) |
| Personal Income | 0.08 *** | 0.08 *** | 0.08 | 0.08 *** |
|  | (0.06 – 0.10) | (0.06 – 0.10) | (0.06 – 0.10) | (0.06 – 0.10) |
| Unemployment | 0.07 *** | 0.07 *** | 0.07 *** | 0.07 *** |
|  | (0.05 – 0.09) | (0.05 – 0.09) | (0.05 – 0.09) | (0.05 – 0.09) |
| Income Inequality x Time |  | 0.02 *** |  |  |
|  |  | (0.02 – 0.03) |  |  |
| Personal Income x Time |  |  | 0.03 *** |  |
|  |  |  | (0.03 – 0.04) |  |
| Education x Time |  |  |  | 0.04 *** |
|  |  |  |  | (0.04 – 0.05) |
| **Random Effects** |  |  |  |  |
| σ2 | 0.5 | 0.5 | 0.5 | 0.5 |
| τ00 | 0.13 county | 0.13 county | 0.13 county | 0.13 county |
|  | 0.07 state | 0.07 state | 0.07 state | 0.07 state |
| ICC | 0.28 | 0.28 | 0.28 | 0.28 |
| N | 2997 county | 2997 county | 2997 county | 2997 county |
|  | 51 state | 51 state | 51 state | 51 state |
| Observations | 104895 | 104895 | 104895 | 104895 |
| Marginal R2 / Conditional R2 | 0.316 / 0.510 | 0.317 / 0.511 | 0.318 / 0.512 | 0.318 / 0.512 |

*Note:* **p* < 0.05, ***p* < 0.01, ****p* < 0.001; 95% Confidence Intervals given in parentheses

Table B. Social Distancing (Reduction in Mobility) from March 08 to April 12 (Psychological & Cultural)

|  | **1** | **2** | **3** | **4** | **5** |
| --- | --- | --- | --- | --- | --- |
| **Predictors** | **β** | **β** | **β** | **β** | **β** |
| Intercept | -0.02 *** | -0.02 *** | -0.02 *** | -0.02 *** | -0.02 *** |
|  | (-0.09 – 0.06) | (-0.09 – 0.05) | (-0.09 – 0.06) | (-0.09 – 0.06) | (-0.09 – 0.05) |
| Time | 0.55 *** | 0.55 *** | 0.55 *** | 0.54 *** | 0.55 *** |
|  | (0.54 – 0.55) | (0.55 – 0.56) | (0.54 – 0.55) | (0.54 – 0.55) | (0.55 – 0.56) |
| Shelter-in-Place Order | 0.04 *** | 0.04 *** | 0.04 *** | 0.05 *** | 0.03 *** |
|  | (0.04 – 0.05) | (0.03 – 0.04) | (0.04 – 0.05) | (0.04 – 0.05) | (0.03 – 0.04) |
| Population Density | 0.01 | 0.01 | 0.01 | 0.01 | 0.01 |
|  | (-0.00 – 0.03) | (-0.00 – 0.03) | (-0.00 – 0.03) | (-0.00 – 0.03) | (-0.00 – 0.03) |
| County Population | 0.06 *** | 0.06 *** | 0.06 *** | 0.06 *** | 0.06 *** |
|  | (0.04 – 0.08) | (0.04 – 0.08) | (0.04 – 0.08) | (0.04 – 0.08) | (0.04 – 0.08) |
| Elder (60+) Proportion | 0.03 *** | 0.03 *** | 0.03 *** | 0.03 *** | 0.03 *** |
|  | (0.01 – 0.05) | (0.01 – 0.05) | (0.01 – 0.05) | (0.01 – 0.05) | (0.01 – 0.05) |
| Education Attainment | 0.14 *** | 0.14 *** | 0.14 *** | 0.14 *** | 0.14 *** |
|  | (0.12 – 0.17) | (0.12 – 0.17) | (0.12 – 0.17) | (0.12 – 0.17) | (0.12 – 0.17) |
| Income Inequality | -0.02 * | -0.02 * | -0.02 * | -0.02 * | -0.02 * |
|  | (-0.04 – -0.00) | (-0.04 – -0.00) | (-0.04 – -0.00) | (-0.04 – -0.00) | (-0.04 – -0.00) |
| Personal Income | 0.09 *** | 0.09 *** | 0.09 *** | 0.09 *** | 0.09 *** |
|  | (0.06 – 0.11) | (0.06 – 0.11) | (0.06 – 0.11) | (0.06 – 0.11) | (0.06 – 0.11) |
| Unemployment | 0.06 *** | 0.06 *** | 0.06 *** | 0.06 *** | 0.06 *** |
|  | (0.03 – 0.08) | (0.03 – 0.08) | (0.03 – 0.08) | (0.03 – 0.08) | (0.03 – 0.08) |
| Democrat Proportion | 0.02 * | 0.02 *** | 0.02 * | 0.02 * | 0.02 * |
|  | (0.00 – 0.05) | (0.00 – 0.05) | (0.00 – 0.05) | (0.00 – 0.05) | (0.00 – 0.05) |
| Big 5 Neuroticism | 0.14 *** | 0.14 *** | 0.14 *** | 0.14 *** | 0.14 *** |
|  | (0.07 – 0.20) | (0.07 – 0.20) | (0.07 – 0.20) | (0.07 – 0.20) | (0.07 – 0.20) |
| Tightness-Looseness | -0.04 | -0.04 | -0.04 | -0.03 * | -0.04 |
|  | (-0.11 – 0.04) | (-0.11 – 0.03) | (-0.11 – 0.04) | (-0.11 – 0.04) | (-0.11 – 0.03) |
| Collectivism | -0.01 | -0.01 | -0.01 | -0.01 | -0.01 ** |
|  | (-0.09 – 0.06) | (-0.09 – 0.06) | (-0.09 – 0.06) | (-0.09 – 0.06) | (-0.09 – 0.06) |
| Democrat x Time |  | 0.06 *** |  |  |  |
|  |  | (0.06 – 0.06) |  |  |  |
| Neuroticism x Time |  |  | -0.01 ** |  |  |
|  |  |  | (-0.01 – -0.00) |  |  |
| Tightness x Time |  |  |  | 0.03 *** |  |
|  |  |  |  | (0.03 – 0.03) |  |
| Collectivism x Time |  |  |  |  | 0.06 *** |
|  |  |  |  |  | (0.05 – 0.06) |
| **Random Effects** |  |  |  |  |  |
| σ2 | 0.32 | 0.31 | 0.32 | 0.32 | 0.31 |
| τ00 | 0.13 county | 0.13 county | 0.13 county | 0.13 county | 0.13 county |
|  | 0.04 state | 0.04 state | 0.04 state | 0.04 state | 0.04 state |
| ICC | 0.36 | 0.36 | 0.36 | 0.36 | 0.36 |
| N | 2977 county | 2977 county | 2977 county | 2977 county | 2977 county |
|  | 48 state | 48 state | 48 state | 48 state | 48 state |
| Observations | 104195 | 104195 | 104195 | 104195 | 104195 |
| Marginal R2 / Conditional R2 | 0.407 / 0.618 | 0.410 / 0.622 | 0.407 / 0.618 | 0.408 / 0.619 | 0.410 / 0.622 |

*Note:* **p* < 0.05, ***p* < 0.01, ****p* < 0.001; 95% Confidence Intervals given in parentheses

Table C. Social Distancing (Reduction in Mobility) from March 08 to April 12 (Health)

|  | **1** | **2** | **3** | **4** | **5** |
| --- | --- | --- | --- | --- | --- |
| **Predictors** | **β** | **β** | **β** | **β** | **β** |
| Intercept | 0.01 *** | 0.01 *** | 0.01 *** | 0.01 *** | 0.01 *** |
|  | (-0.06 – 0.08) | (-0.06 – 0.08) | (-0.06 – 0.08) | (-0.06 – 0.09) | (-0.06 – 0.08) |
| Time | 0.55 *** | 0.55 *** | 0.55 *** | 0.56 *** | 0.55 *** |
|  | (0.54 – 0.55) | (0.54 – 0.55) | (0.54 – 0.55) | (0.56 – 0.57) | (0.54 – 0.55) |
| Shelter-in-Place Order | 0.04 *** | 0.04 *** | 0.04 *** | 0.02 *** | 0.04 *** |
|  | (0.04 – 0.05) | (0.04 – 0.05) | (0.04 – 0.05) | (0.01 – 0.03) | (0.04 – 0.05) |
| Population Density | 0.02 | 0.02 | 0.02 | 0.02 | 0.02 |
|  | (-0.00 – 0.03) | (-0.00 – 0.03) | (-0.00 – 0.03) | (-0.00 – 0.03) | (-0.00 – 0.03) |
| County Population | 0.06 *** | 0.06 *** | 0.06 *** | 0.06 *** | 0.06 *** |
|  | (0.05 – 0.08) | (0.05 – 0.08) | (0.05 – 0.08) | (0.05 – 0.08) | (0.05 – 0.08) |
| Elder (60+) Proportion | 0.02 ** | 0.02 ** | 0.02 ** | 0.02 ** | 0.02 ** |
|  | (0.01 – 0.04) | (0.01 – 0.04) | (0.01 – 0.04) | (0.01 – 0.04) | (0.01 – 0.04) |
| Education Attainment | 0.13 *** | 0.13 *** | 0.13 *** | 0.13 *** | 0.13 *** |
|  | (0.10 – 0.16) | (0.10 – 0.16) | (0.10 – 0.16) | (0.10 – 0.16) | (0.10 – 0.16) |
| Income Inequality | -0.01 | -0.01 | -0.01 | -0.01 | -0.01 |
|  | (-0.03 – 0.01) | (-0.03 – 0.01) | (-0.03 – 0.01) | (-0.03 – 0.01) | (-0.03 – 0.01) |
| Personal Income | 0.09 *** | 0.09 *** | 0.09 *** | 0.09 *** | 0.09 *** |
|  | (0.07 – 0.11) | (0.07 – 0.11) | (0.07 – 0.11) | (0.07 – 0.11) | (0.07 – 0.11) |
| Unemployment | 0.08 *** | 0.08 *** | 0.08 *** | 0.08 *** | 0.08 *** |
|  | (0.05 – 0.10) | (0.05 – 0.10) | (0.05 – 0.10) | (0.05 – 0.10) | (0.05 – 0.10) |
| Preventable Hospitalization | 0.02 * | 0.02 ** | 0.02 * | 0.02 * | 0.02 * |
|  | (0.00 – 0.04) | (0.00 – 0.04) | (0.00 – 0.04) | (0.00 – 0.04) | (0.00 – 0.04) |
| Vaccination | 0.01 | 0.01 | 0.01 *** | 0.01 | 0.01 |
|  | (-0.01 – 0.03) | (-0.01 – 0.03) | (-0.01 – 0.03) | (-0.01 – 0.03) | (-0.01 – 0.03) |
| Fair/Poor Health | -0.04 * | -0.04 * | -0.04 * | -0.04 * | -0.04 * |
|  | (-0.07 – -0.00) | (-0.07 – -0.00) | (-0.07 – -0.00) | (-0.07 – -0.00) | (-0.07 – -0.00) |
| At-Risk Preparedness Index | 0.09 ** | 0.09 ** | 0.09 ** | 0.09 | 0.09 ** |
|  | (0.03 – 0.14) | (0.03 – 0.14) | (0.03 – 0.14) | (0.04 – 0.14) | (0.03 – 0.14) |
| Surveillance & Epidemiological Index | 0.02 | 0.02 | 0.02 | 0.02 | 0.02 |
|  | (-0.05 – 0.09) | (-0.05 – 0.09) | (-0.05 – 0.09) | (-0.05 – 0.09) | (-0.05 – 0.09) |
| Hospitalization x Time |  | -0.00 * |  |  |  |
|  |  | (-0.01 – -0.00) |  |  |  |
| Vaccination x Time |  |  | 0.03 *** |  |  |
|  |  |  | (0.03 – 0.03) |  |  |
| At-Risk x Time |  |  |  | 0.06 *** |  |
|  |  |  |  | (0.06 – 0.06) |  |
| Surveillance x Time |  |  |  |  | -0.03 *** |
|  |  |  |  |  | (-0.03 – -0.02) |
| **Random Effects** |  |  |  |  |  |
| σ2 | 0.32 | 0.32 | 0.32 | 0.32 | 0.32 |
| τ00 | 0.13 county | 0.13 county | 0.13 county | 0.13 county | 0.13 county |
|  | 0.05 state | 0.05 state | 0.05 state | 0.05 state | 0.05 state |
| ICC | 0.36 | 0.36 | 0.36 | 0.36 | 0.36 |
| N | 2987 county | 2987 county | 2987 county | 2987 county | 2987 county |
|  | 51 state | 51 state | 51 state | 51 state | 51 state |
| Observations | 104545 | 104545 | 104545 | 104545 | 104545 |
| Marginal R2 / Conditional R2 | 0.399 / 0.613 | 0.399 / 0.613 | 0.399 / 0.614 | 0.401 / 0.617 | 0.399 / 0.614 |

*Note:* **p* < 0.05, ***p* < 0.01, ****p* < 0.001; 95% Confidence Intervals given in parentheses

**1.2 Change in Non-Essential Visitation by Category of Variables for Time Frame 1**

Table D. Social Distancing (Reduction in Non-Essential Visitation) from March 08 to April 12 (SES)

|  | **1** | **2** | **3** | **4** |
| --- | --- | --- | --- | --- |
| **Predictors** | **β** | **β** | **β** | **β** |
| Intercept | 0.01 *** | 0.01 *** | 0.01 *** | 0.01 *** |
|  | (-0.04 – 0.06) | (-0.04 – 0.06) | (-0.04 – 0.06) | (-0.04 – 0.06) |
| Time | 0.63 *** | 0.63 *** | 0.63 *** | 0.63 *** |
|  | (0.62 – 0.64) | (0.62 – 0.64) | (0.62 – 0.64) | (0.62 – 0.64) |
| Shelter-in-Place Order | -0.06 *** | -0.06 *** | -0.06 *** | -0.06 *** |
|  | (-0.07 – -0.06) | (-0.07 – -0.06) | (-0.07 – -0.06) | (-0.07 – -0.06) |
| Population Density | 0.00 | 0.00 | 0.00 | 0.00 |
|  | (-0.01 – 0.02) | (-0.01 – 0.02) | (-0.01 – 0.02) | (-0.01 – 0.02) |
| County Population | 0.04 *** | 0.04 *** | 0.04 *** | 0.04 *** |
|  | (0.02 – 0.05) | (0.02 – 0.05) | (0.02 – 0.05) | (0.02 – 0.05) |
| Elder (60+) Proportion | -0.11 *** | -0.11 *** | -0.11 *** | -0.11 *** |
|  | (-0.12 – -0.09) | (-0.12 – -0.09) | (-0.12 – -0.09) | (-0.12 – -0.09) |
| Education Attainment | 0.26 *** | 0.26 *** | 0.26 *** | 0.26 *** |
|  | (0.24 – 0.29) | (0.24 – 0.29) | (0.24 – 0.29) | (0.24 – 0.29) |
| Income Inequality | 0.09 *** | 0.09 *** | 0.09 *** | 0.09 *** |
|  | (0.07 – 0.11) | (0.07 – 0.11) | (0.07 – 0.11) | (0.07 – 0.11) |
| Personal Income | 0.00 | 0.00 | 0.00 *** | 0.00 |
|  | (-0.02 – 0.02) | (-0.02 – 0.02) | (-0.02 – 0.03) | (-0.02 – 0.02) |
| Unemployment | 0.02 | 0.02 | 0.02 | 0.02 |
|  | (-0.00 – 0.04) | (-0.00 – 0.04) | (-0.00 – 0.04) | (-0.00 – 0.04) |
| Income Inequality x Time |  | 0.01 *** |  |  |
|  |  | (0.00 – 0.01) |  |  |
| Personal Income x Time |  |  | 0.04 *** |  |
|  |  |  | (0.03 – 0.04) |  |
| Education x Time |  |  |  | 0.04 *** |
|  |  |  |  | (0.04 – 0.05) |
| **Random Effects** |  |  |  |  |
| σ2 | 0.29 | 0.29 | 0.29 | 0.29 |
| τ00 | 0.07 county | 0.07 county | 0.07 county | 0.07 county |
|  | 0.02 state | 0.02 state | 0.02 state | 0.02 state |
| ICC | 0.23 | 0.23 | 0.24 | 0.24 |
| N | 2028 county | 2028 county | 2028 county | 2028 county |
|  | 51 state | 51 state | 51 state | 51 state |
| Observations | 70976 | 70976 | 70976 | 70976 |
| Marginal R2 / Conditional R2 | 0.441 / 0.572 | 0.441 / 0.572 | 0.442 / 0.573 | 0.443 / 0.574 |

*Note:* **p* < 0.05, ***p* < 0.01, ****p* < 0.001; 95% Confidence Intervals given in parentheses

Table E. Social Distancing (Reduction in Non-Essential Visitation) from March 08 to April 12 (Psychological & Cultural)

|  | **1** | **2** | **3** | **4** | **5** |
| --- | --- | --- | --- | --- | --- |
| **Predictors** | **β** | **β** | **β** | **β** | **β** |
| Intercept | -0.01 *** | -0.02 *** | -0.01 *** | -0.02 *** | -0.02 *** |
|  | (-0.05 – 0.02) | (-0.05 – 0.02) | (-0.05 – 0.02) | (-0.05 – 0.02) | (-0.05 – 0.02) |
| Time | 0.63 *** | 0.63 *** | 0.63 *** | 0.63 *** | 0.63 *** |
|  | (0.62 – 0.64) | (0.62 – 0.64) | (0.62 – 0.64) | (0.62 – 0.64) | (0.62 – 0.64) |
| Shelter-in-Place Order | -0.06 *** | -0.07 *** | -0.06 *** | -0.07 *** | -0.07 *** |
|  | (-0.07 – -0.06) | (-0.08 – -0.06) | (-0.07 – -0.05) | (-0.07 – -0.06) | (-0.07 – -0.06) |
| Population Density | -0.00 | -0.00 | -0.00 | -0.00 | -0.00 |
|  | (-0.02 – 0.02) | (-0.02 – 0.02) | (-0.02 – 0.02) | (-0.02 – 0.02) | (-0.02 – 0.02) |
| County Population | 0.03 ** | 0.03 ** | 0.03 ** | 0.03 ** | 0.03 ** |
|  | (0.01 – 0.04) | (0.01 – 0.04) | (0.01 – 0.04) | (0.01 – 0.04) | (0.01 – 0.04) |
| Elder (60+) Proportion | -0.09 *** | -0.09 *** | -0.09 *** | -0.09 *** | -0.09 *** |
|  | (-0.11 – -0.08) | (-0.11 – -0.08) | (-0.11 – -0.08) | (-0.11 – -0.08) | (-0.11 – -0.08) |
| Education Attainment | 0.24 *** | 0.24 *** | 0.24 *** | 0.24 *** | 0.24 *** |
|  | (0.22 – 0.27) | (0.22 – 0.27) | (0.22 – 0.27) | (0.22 – 0.27) | (0.22 – 0.27) |
| Income Inequality | 0.07 *** | 0.07 *** | 0.07 *** | 0.07 *** | 0.07 *** |
|  | (0.05 – 0.09) | (0.05 – 0.09) | (0.05 – 0.09) | (0.05 – 0.09) | (0.05 – 0.09) |
| Personal Income | -0.01 | -0.01 | -0.01 | -0.01 | -0.01 |
|  | (-0.03 – 0.02) | (-0.03 – 0.02) | (-0.03 – 0.02) | (-0.03 – 0.02) | (-0.03 – 0.02) |
| Unemployment | 0.01 | 0.01 | 0.01 | 0.01 | 0.01 |
|  | (-0.01 – 0.03) | (-0.01 – 0.03) | (-0.01 – 0.03) | (-0.01 – 0.03) | (-0.01 – 0.03) |
| Democrat Proportion | 0.08 *** | 0.08 | 0.08 *** | 0.08 *** | 0.08 *** |
|  | (0.05 – 0.10) | (0.05 – 0.10) | (0.05 – 0.10) | (0.05 – 0.10) | (0.05 – 0.10) |
| Big 5 Neuroticism | 0.06 *** | 0.06 *** | 0.06 *** | 0.06 *** | 0.06 *** |
|  | (0.03 – 0.09) | (0.03 – 0.10) | (0.03 – 0.09) | (0.03 – 0.09) | (0.03 – 0.09) |
| Tightness-Looseness | -0.09 *** | -0.09 *** | -0.09 *** | -0.09 *** | -0.09 *** |
|  | (-0.13 – -0.05) | (-0.13 – -0.05) | (-0.13 – -0.05) | (-0.13 – -0.05) | (-0.13 – -0.05) |
| Collectivism | 0.02 | 0.02 | 0.02 | 0.02 | 0.02 |
|  | (-0.02 – 0.06) | (-0.01 – 0.06) | (-0.02 – 0.06) | (-0.02 – 0.06) | (-0.02 – 0.06) |
| Democrat x Time |  | 0.04 *** |  |  |  |
|  |  | (0.04 – 0.05) |  |  |  |
| Neuroticism x Time |  |  | -0.02 *** |  |  |
|  |  |  | (-0.02 – -0.01) |  |  |
| Tightness x Time |  |  |  | -0.01 ** |  |
|  |  |  |  | (-0.01 – -0.00) |  |
| Collectivism x Time |  |  |  |  | 0.01 *** |
|  |  |  |  |  | (0.01 – 0.02) |
| **Random Effects** |  |  |  |  |  |
| σ2 | 0.29 | 0.29 | 0.29 | 0.29 | 0.29 |
| τ00 | 0.07 county | 0.07 county | 0.07 county | 0.07 county | 0.07 county |
|  | 0.01 state | 0.01 state | 0.01 state | 0.01 state | 0.01 state |
| ICC | 0.2 | 0.2 | 0.2 | 0.2 | 0.2 |
| N | 2019 county | 2019 county | 2019 county | 2019 county | 2019 county |
|  | 48 state | 48 state | 48 state | 48 state | 48 state |
| Observations | 70661 | 70661 | 70661 | 70661 | 70661 |
| Marginal R2 / Conditional R2 | 0.463 / 0.572 | 0.465 / 0.574 | 0.464 / 0.573 | 0.463 / 0.572 | 0.463 / 0.573 |

*Note:* **p* < 0.05, ***p* < 0.01, ****p* < 0.001; 95% Confidence Intervals given in parentheses

Table F. Social Distancing (Reduction in Non-Essential Visitation) from March 08 to April 12 (Health)

|  | **1** | **2** | **3** | **4** | **5** |
| --- | --- | --- | --- | --- | --- |
| **Predictors** | **β** | **β** | **β** | **β** | **β** |
| Intercept | 0.03 *** | 0.03 *** | 0.03 *** | 0.03 *** | 0.03 *** |
|  | (-0.02 – 0.08) | (-0.02 – 0.08) | (-0.02 – 0.08) | (-0.02 – 0.08) | (-0.02 – 0.08) |
| Time | 0.63 *** | 0.63 *** | 0.63 *** | 0.63 *** | 0.63 *** |
|  | (0.62 – 0.64) | (0.62 – 0.64) | (0.62 – 0.64) | (0.63 – 0.64) | (0.62 – 0.64) |
| Shelter-in-Place Order | -0.06 *** | -0.06 *** | -0.06 *** | -0.07 *** | -0.06 *** |
|  | (-0.07 – -0.05) | (-0.07 – -0.05) | (-0.07 – -0.05) | (-0.08 – -0.06) | (-0.07 – -0.05) |
| Population Density | 0.01 | 0.01 | 0.01 | 0.01 | 0.01 |
|  | (-0.01 – 0.02) | (-0.01 – 0.02) | (-0.01 – 0.02) | (-0.01 – 0.02) | (-0.01 – 0.02) |
| County Population | 0.04 *** | 0.04 *** | 0.04 *** | 0.04 *** | 0.04 *** |
|  | (0.02 – 0.05) | (0.02 – 0.05) | (0.02 – 0.05) | (0.02 – 0.05) | (0.02 – 0.05) |
| Elder (60+) Proportion | -0.09 *** | -0.09 *** | -0.09 *** | -0.09 *** | -0.09 *** |
|  | (-0.11 – -0.07) | (-0.11 – -0.07) | (-0.11 – -0.07) | (-0.11 – -0.07) | (-0.11 – -0.07) |
| Education Attainment | 0.28 *** | 0.28 *** | 0.28 *** | 0.28 *** | 0.28 *** |
|  | (0.25 – 0.31) | (0.25 – 0.31) | (0.25 – 0.31) | (0.25 – 0.31) | (0.25 – 0.31) |
| Income Inequality | 0.06 *** | 0.06 *** | 0.06 *** | 0.06 *** | 0.06 *** |
|  | (0.04 – 0.09) | (0.04 – 0.09) | (0.04 – 0.09) | (0.04 – 0.09) | (0.04 – 0.09) |
| Personal Income | 0.01 | 0.01 | 0.01 | 0.01 | 0.01 |
|  | (-0.02 – 0.03) | (-0.02 – 0.03) | (-0.02 – 0.03) | (-0.02 – 0.03) | (-0.02 – 0.03) |
| Unemployment | 0.01 | 0.01 | 0.01 | 0.01 | 0.01 |
|  | (-0.01 – 0.03) | (-0.01 – 0.03) | (-0.01 – 0.03) | (-0.01 – 0.03) | (-0.01 – 0.03) |
| Preventable Hospitalization | -0.02 | -0.02 | -0.02 | -0.02 | -0.02 |
|  | (-0.04 – 0.00) | (-0.04 – 0.00) | (-0.04 – 0.00) | (-0.04 – 0.00) | (-0.04 – 0.00) |
| Vaccination | 0.07 *** | 0.07 *** | 0.07 *** | 0.07 *** | 0.07 *** |
|  | (0.05 – 0.09) | (0.05 – 0.09) | (0.05 – 0.09) | (0.05 – 0.09) | (0.05 – 0.09) |
| Fair/Poor Health | 0.11 *** | 0.11 *** | 0.11 *** | 0.11 *** | 0.11 *** |
|  | (0.07 – 0.14) | (0.07 – 0.14) | (0.07 – 0.14) | (0.07 – 0.14) | (0.07 – 0.14) |
| At-Risk Preparedness Index | 0.06 *** | 0.06 *** | 0.06 *** | 0.06 | 0.06 *** |
|  | (0.02 – 0.09) | (0.02 – 0.09) | (0.02 – 0.09) | (0.02 – 0.09) | (0.02 – 0.09) |
| Surveillance & Epidemiological Index | -0.00 | -0.00 | -0.00 | -0.00 | -0.00 |
|  | (-0.05 – 0.04) | (-0.05 – 0.04) | (-0.05 – 0.04) | (-0.05 – 0.04) | (-0.05 – 0.04) |
| Hospitalization x Time |  | -0.01 *** |  |  |  |
|  |  | (-0.02 – -0.01) |  |  |  |
| Vaccination x Time |  |  | 0.00 |  |  |
|  |  |  | (-0.00 – 0.01) |  |  |
| At-Risk x Time |  |  |  | 0.02 *** |  |
|  |  |  |  | (0.02 – 0.03) |  |
| Surveillance x Time |  |  |  |  | -0.01 ** |
|  |  |  |  |  | (-0.01 – -0.00) |
| **Random Effects** |  |  |  |  |  |
| σ2 | 0.29 | 0.29 | 0.29 | 0.29 | 0.29 |
| τ00 | 0.06 county | 0.06 county | 0.06 county | 0.06 county | 0.06 county |
|  | 0.02 state | 0.02 state | 0.02 state | 0.02 state | 0.02 state |
| ICC | 0.22 | 0.22 | 0.22 | 0.22 | 0.22 |
| N | 2025 county | 2025 county | 2025 county | 2025 county | 2025 county |
|  | 51 state | 51 state | 51 state | 51 state | 51 state |
| Observations | 70872 | 70872 | 70872 | 70872 | 70872 |
| Marginal R2 / Conditional R2 | 0.447 / 0.569 | 0.447 / 0.569 | 0.447 / 0.569 | 0.447 / 0.570 | 0.447 / 0.569 |

*Note:* **p* < 0.05, ***p* < 0.01, ****p* < 0.001; 95% Confidence Intervals given in parentheses

**1.3 Change in Distance by Category of Variables for Time Frame 2**

Table G. Social Distancing (Reduction in Mobility) from April 13 to May 24 (SES)

|  | **1** | **2** | **3** | **4** |
| --- | --- | --- | --- | --- |
| **Predictors** | **β** | **β** | **β** | **β** |
| Intercept | 0.04 | 0.04 | 0.04 | 0.04 |
|  | (-0.06 – 0.14) | (-0.06 – 0.14) | (-0.06 – 0.14) | (-0.06 – 0.14) |
| Time | -0.51 *** | -0.51 *** | -0.51 *** | -0.51 *** |
|  | (-0.51 – -0.50) | (-0.51 – -0.50) | (-0.51 – -0.50) | (-0.51 – -0.50) |
| Shelter-in-Place Order | 0.09 | 0.09 | 0.09 | 0.09 |
|  | (-0.01 – 0.18) | (-0.01 – 0.18) | (-0.01 – 0.18) | (-0.01 – 0.18) |
| Population Density | 0.02 * | 0.02 * | 0.02 * | 0.02 * |
|  | (0.00 – 0.04) | (0.00 – 0.04) | (0.00 – 0.04) | (0.00 – 0.04) |
| County Population | 0.09 *** | 0.09 *** | 0.09 *** | 0.09 *** |
|  | (0.08 – 0.11) | (0.08 – 0.11) | (0.08 – 0.11) | (0.08 – 0.11) |
| Elder (60+) Proportion | -0.09 *** | -0.09 *** | -0.09 *** | -0.09 *** |
|  | (-0.10 – -0.07) | (-0.10 – -0.07) | (-0.10 – -0.07) | (-0.10 – -0.07) |
| Education Attainment | 0.18 *** | 0.18 *** | 0.18 *** | 0.18 *** |
|  | (0.15 – 0.20) | (0.15 – 0.20) | (0.15 – 0.20) | (0.15 – 0.20) |
| Income Inequality | 0.01 | 0.01 | 0.01 | 0.01 |
|  | (-0.01 – 0.03) | (-0.01 – 0.03) | (-0.01 – 0.03) | (-0.01 – 0.03) |
| Personal Income | 0.14 *** | 0.14 *** | 0.14 * | 0.14 *** |
|  | (0.11 – 0.16) | (0.11 – 0.16) | (0.11 – 0.16) | (0.11 – 0.16) |
| Unemployment | 0.09 *** | 0.09 *** | 0.09 *** | 0.09 *** |
|  | (0.06 – 0.11) | (0.06 – 0.11) | (0.06 – 0.11) | (0.06 – 0.11) |
| Income Inequality x Time |  | -0.00 |  |  |
|  |  | (-0.00 – 0.00) |  |  |
| Personal Income x Time |  |  | 0.02 *** |  |
|  |  |  | (0.02 – 0.03) |  |
| Education x Time |  |  |  | 0.01 *** |
|  |  |  |  | (0.00 – 0.01) |
| **Random Effects** |  |  |  |  |
| σ2 | 0.37 | 0.37 | 0.37 | 0.37 |
| τ00 | 0.19 county | 0.19 county | 0.19 county | 0.19 county |
|  | 0.12 state | 0.12 state | 0.12 state | 0.12 state |
| ICC | 0.46 | 0.46 | 0.46 | 0.46 |
| N | 2997 county | 2997 county | 2997 county | 2997 county |
|  | 51 state | 51 state | 51 state | 51 state |
| Observations | 131868 | 131868 | 131868 | 131868 |
| Marginal R2 / Conditional R2 | 0.353 / 0.650 | 0.353 / 0.650 | 0.354 / 0.651 | 0.353 / 0.650 |

*Note:* **p* < 0.05, ***p* < 0.01, ****p* < 0.001; 95% Confidence Intervals given in parentheses

Table H. Social Distancing (Reduction in Mobility) from April 13 to May 24 (Psychological & Cultural)

|  | **1** | **2** | **3** | **4** | **5** |
| --- | --- | --- | --- | --- | --- |
| **Predictors** | **β** | **β** | **β** | **β** | **β** |
| Intercept | -0.02 *** | -0.02 ** | -0.02 | -0.02 ** | -0.02 * |
|  | (-0.08 – 0.04) | (-0.08 – 0.04) | (-0.08 – 0.04) | (-0.08 – 0.04) | (-0.08 – 0.04) |
| Time | -0.51 *** | -0.51 *** | -0.51 *** | -0.51 *** | -0.51 *** |
|  | (-0.51 – -0.51) | (-0.51 – -0.51) | (-0.51 – -0.51) | (-0.51 – -0.51) | (-0.51 – -0.51) |
| Shelter-in-Place Order | 0.04 | 0.04 | 0.04 | 0.04 | 0.04 |
|  | (-0.02 – 0.10) | (-0.02 – 0.10) | (-0.02 – 0.10) | (-0.02 – 0.10) | (-0.02 – 0.10) |
| Population Density | 0.01 | 0.01 | 0.01 | 0.01 | 0.01 |
|  | (-0.00 – 0.03) | (-0.00 – 0.03) | (-0.00 – 0.03) | (-0.00 – 0.03) | (-0.00 – 0.03) |
| County Population | 0.07 *** | 0.07 *** | 0.07 *** | 0.07 *** | 0.07 *** |
|  | (0.05 – 0.09) | (0.05 – 0.09) | (0.05 – 0.09) | (0.05 – 0.09) | (0.05 – 0.09) |
| Elder (60+) Proportion | -0.05 *** | -0.05 *** | -0.05 *** | -0.05 *** | -0.05 *** |
|  | (-0.07 – -0.03) | (-0.07 – -0.03) | (-0.07 – -0.03) | (-0.07 – -0.03) | (-0.07 – -0.03) |
| Education Attainment | 0.14 *** | 0.14 *** | 0.14 *** | 0.14 *** | 0.14 *** |
|  | (0.12 – 0.17) | (0.12 – 0.17) | (0.12 – 0.17) | (0.12 – 0.17) | (0.12 – 0.17) |
| Income Inequality | -0.03 ** | -0.03 ** | -0.03 ** | -0.03 ** | -0.03 ** |
|  | (-0.05 – -0.01) | (-0.05 – -0.01) | (-0.05 – -0.01) | (-0.05 – -0.01) | (-0.05 – -0.01) |
| Personal Income | 0.12 *** | 0.12 *** | 0.12 *** | 0.12 *** | 0.12 *** |
|  | (0.09 – 0.14) | (0.09 – 0.14) | (0.09 – 0.14) | (0.09 – 0.14) | (0.09 – 0.14) |
| Unemployment | 0.03 * | 0.03 * | 0.03 * | 0.03 * | 0.03 * |
|  | (0.01 – 0.06) | (0.01 – 0.06) | (0.01 – 0.06) | (0.01 – 0.06) | (0.01 – 0.06) |
| Democrat Proportion | 0.16 *** | 0.16 *** | 0.16 *** | 0.16 *** | 0.16 *** |
|  | (0.14 – 0.18) | (0.14 – 0.18) | (0.14 – 0.18) | (0.14 – 0.18) | (0.14 – 0.18) |
| Big 5 Neuroticism | 0.19 *** | 0.19 *** | 0.19 | 0.19 *** | 0.19 *** |
|  | (0.14 – 0.25) | (0.14 – 0.25) | (0.14 – 0.25) | (0.14 – 0.25) | (0.14 – 0.25) |
| Tightness-Looseness | 0.03 | 0.03 | 0.03 | 0.03 | 0.03 |
|  | (-0.04 – 0.09) | (-0.04 – 0.09) | (-0.04 – 0.09) | (-0.04 – 0.09) | (-0.04 – 0.09) |
| Collectivism | -0.03 | -0.03 | -0.03 | -0.03 | -0.03 ** |
|  | (-0.10 – 0.03) | (-0.10 – 0.03) | (-0.10 – 0.03) | (-0.10 – 0.03) | (-0.10 – 0.03) |
| Democrat x Time |  | 0.02 *** |  |  |  |
|  |  | (0.02 – 0.03) |  |  |  |
| Neuroticism x Time |  |  | 0.03 *** |  |  |
|  |  |  | (0.03 – 0.03) |  |  |
| Tightness x Time |  |  |  | 0.01 ** |  |
|  |  |  |  | (0.00 – 0.01) |  |
| Collectivism x Time |  |  |  |  | 0.02 *** |
|  |  |  |  |  | (0.01 – 0.02) |
| **Random Effects** |  |  |  |  |  |
| σ2 | 0.36 | 0.36 | 0.36 | 0.36 | 0.36 |
| τ00 | 0.18 county | 0.18 county | 0.18 county | 0.18 county | 0.18 county |
|  | 0.04 state | 0.04 state | 0.04 state | 0.04 state | 0.04 state |
| ICC | 0.37 | 0.37 | 0.37 | 0.37 | 0.37 |
| N | 2977 county | 2977 county | 2977 county | 2977 county | 2977 county |
|  | 48 state | 48 state | 48 state | 48 state | 48 state |
| Observations | 130988 | 130988 | 130988 | 130988 | 130988 |
| Marginal R2 / Conditional R2 | 0.416 / 0.634 | 0.417 / 0.634 | 0.417 / 0.635 | 0.416 / 0.634 | 0.416 / 0.634 |

*Note:* **p* < 0.05, ***p* < 0.01, ****p* < 0.001; 95% Confidence Intervals given in parentheses

Table I. Social Distancing (Reduction in Mobility) from April 13 to May 24 (Health)

|  | **1** | **2** | **3** | **4** | **5** |
| --- | --- | --- | --- | --- | --- |
| **Predictors** | **β** | **β** | **β** | **β** | **β** |
| Intercept | 0.04 *** | 0.04 *** | 0.04 * | 0.04 ** | 0.04 *** |
|  | (-0.06 – 0.13) | (-0.06 – 0.13) | (-0.06 – 0.13) | (-0.06 – 0.13) | (-0.06 – 0.13) |
| Time | -0.51 *** | -0.51 *** | -0.51 *** | -0.51 *** | -0.51 *** |
|  | (-0.51 – -0.51) | (-0.51 – -0.51) | (-0.51 – -0.51) | (-0.51 – -0.51) | (-0.51 – -0.51) |
| Shelter-in-Place Order | 0.03 | 0.03 | 0.03 | 0.03 | 0.03 |
|  | (-0.07 – 0.12) | (-0.07 – 0.12) | (-0.07 – 0.12) | (-0.07 – 0.12) | (-0.07 – 0.12) |
| Population Density | 0.02 * | 0.02 * | 0.02 * | 0.02 * | 0.02 * |
|  | (0.00 – 0.04) | (0.00 – 0.04) | (0.00 – 0.04) | (0.00 – 0.04) | (0.00 – 0.04) |
| County Population | 0.09 *** | 0.09 *** | 0.09 *** | 0.09 *** | 0.09 *** |
|  | (0.07 – 0.11) | (0.07 – 0.11) | (0.07 – 0.11) | (0.07 – 0.11) | (0.07 – 0.11) |
| Elder (60+) Proportion | -0.07 *** | -0.07 *** | -0.07 *** | -0.07 *** | -0.07 *** |
|  | (-0.09 – -0.05) | (-0.09 – -0.05) | (-0.09 – -0.05) | (-0.09 – -0.05) | (-0.09 – -0.05) |
| Education Attainment | 0.20 *** | 0.20 *** | 0.20 *** | 0.20 *** | 0.20 *** |
|  | (0.17 – 0.23) | (0.17 – 0.23) | (0.17 – 0.23) | (0.17 – 0.23) | (0.17 – 0.23) |
| Income Inequality | -0.01 | -0.01 | -0.01 | -0.01 | -0.01 |
|  | (-0.03 – 0.01) | (-0.03 – 0.01) | (-0.03 – 0.01) | (-0.03 – 0.01) | (-0.03 – 0.01) |
| Personal Income | 0.14 *** | 0.14 *** | 0.14 *** | 0.14 *** | 0.14 *** |
|  | (0.12 – 0.17) | (0.12 – 0.17) | (0.12 – 0.17) | (0.12 – 0.17) | (0.12 – 0.17) |
| Unemployment | 0.07 *** | 0.07 *** | 0.07 *** | 0.07 *** | 0.07 *** |
|  | (0.05 – 0.10) | (0.05 – 0.10) | (0.05 – 0.10) | (0.05 – 0.10) | (0.05 – 0.10) |
| Preventable Hospitalization | 0.00 | 0.00 | 0.00 | 0.00 | 0.00 |
|  | (-0.02 – 0.02) | (-0.02 – 0.02) | (-0.02 – 0.02) | (-0.02 – 0.02) | (-0.02 – 0.02) |
| Vaccination | 0.03 * | 0.03 * | 0.03 *** | 0.03 * | 0.03 * |
|  | (0.01 – 0.05) | (0.01 – 0.05) | (0.01 – 0.05) | (0.01 – 0.05) | (0.01 – 0.05) |
| Fair/Poor Health | 0.08 *** | 0.08 *** | 0.08 *** | 0.08 *** | 0.08 *** |
|  | (0.04 – 0.12) | (0.04 – 0.12) | (0.04 – 0.12) | (0.04 – 0.12) | (0.04 – 0.12) |
| At-Risk Preparedness Index | 0.11 ** | 0.11 ** | 0.11 ** | 0.11 | 0.11 ** |
|  | (0.04 – 0.18) | (0.04 – 0.18) | (0.04 – 0.18) | (0.04 – 0.18) | (0.04 – 0.18) |
| Surveillance & Epidemiological Index | 0.01 | 0.01 | 0.01 | 0.01 | 0.01 |
|  | (-0.08 – 0.10) | (-0.08 – 0.10) | (-0.08 – 0.10) | (-0.08 – 0.10) | (-0.08 – 0.10) |
| Hospitalization x Time |  | 0.00 ** |  |  |  |
|  |  | (0.00 – 0.01) |  |  |  |
| Vaccination x Time |  |  | 0.03 *** |  |  |
|  |  |  | (0.02 – 0.03) |  |  |
| At-Risk x Time |  |  |  | 0.02 *** |  |
|  |  |  |  | (0.01 – 0.02) |  |
| Surveillance x Time |  |  |  |  | 0.00 |
|  |  |  |  |  | (-0.00 – 0.00) |
| **Random Effects** |  |  |  |  |  |
| σ2 | 0.36 | 0.36 | 0.36 | 0.36 | 0.36 |
| τ00 | 0.19 county | 0.19 county | 0.19 county | 0.19 county | 0.19 county |
|  | 0.10 state | 0.10 state | 0.10 state | 0.10 state | 0.10 state |
| ICC | 0.45 | 0.45 | 0.45 | 0.45 | 0.45 |
| N | 2987 county | 2987 county | 2987 county | 2987 county | 2987 county |
|  | 51 state | 51 state | 51 state | 51 state | 51 state |
| Observations | 131428 | 131428 | 131428 | 131428 | 131428 |
| Marginal R2 / Conditional R2 | 0.372 / 0.654 | 0.372 / 0.654 | 0.373 / 0.654 | 0.372 / 0.654 | 0.372 / 0.654 |

*Note:* **p* < 0.05, ***p* < 0.01, ****p* < 0.001; 95% Confidence Intervals given in parentheses

**1.2 Change in Non-Essential Visitation by Category of Variables for Time Frame 2**

Table G. Social Distancing (Reduction in Non-Essential Visitations) from April 13 to May 24 (SES)

|  | **1** | **2** | **3** | **4** |
| --- | --- | --- | --- | --- |
| **Predictors** | **β** | **β** | **β** | **β** |
| Intercept | 0.03 *** | 0.03 | 0.03 *** | 0.03 ** |
|  | (-0.04 – 0.10) | (-0.04 – 0.10) | (-0.04 – 0.10) | (-0.04 – 0.10) |
| Time | -0.43 *** | -0.43 *** | -0.43 *** | -0.43 *** |
|  | (-0.43 – -0.42) | (-0.43 – -0.42) | (-0.43 – -0.42) | (-0.43 – -0.42) |
| Shelter-in-Place Order | 0.07 * | 0.07 * | 0.07 * | 0.07 * |
|  | (0.02 – 0.13) | (0.02 – 0.13) | (0.02 – 0.13) | (0.02 – 0.13) |
| Population Density | 0.01 | 0.01 | 0.01 | 0.01 |
|  | (-0.01 – 0.04) | (-0.01 – 0.04) | (-0.01 – 0.04) | (-0.01 – 0.04) |
| County Population | 0.05 *** | 0.05 *** | 0.05 *** | 0.05 *** |
|  | (0.03 – 0.08) | (0.03 – 0.08) | (0.03 – 0.08) | (0.03 – 0.08) |
| Elder (60+) Proportion | -0.16 *** | -0.16 *** | -0.16 *** | -0.16 *** |
|  | (-0.19 – -0.14) | (-0.19 – -0.14) | (-0.19 – -0.14) | (-0.19 – -0.14) |
| Education Attainment | 0.27 *** | 0.27 *** | 0.27 *** | 0.27 *** |
|  | (0.24 – 0.30) | (0.24 – 0.30) | (0.24 – 0.30) | (0.24 – 0.30) |
| Income Inequality | 0.12 *** | 0.12 | 0.12 *** | 0.12 *** |
|  | (0.09 – 0.14) | (0.09 – 0.14) | (0.09 – 0.14) | (0.09 – 0.14) |
| Personal Income | -0.00 | -0.00 | -0.00 *** | -0.00 |
|  | (-0.03 – 0.03) | (-0.03 – 0.03) | (-0.03 – 0.03) | (-0.03 – 0.03) |
| Unemployment | 0.00 | 0.00 | 0.00 | 0.00 |
|  | (-0.03 – 0.03) | (-0.03 – 0.03) | (-0.03 – 0.03) | (-0.03 – 0.03) |
| Income Inequality x Time |  | 0.03 *** |  |  |
|  |  | (0.03 – 0.04) |  |  |
| Personal Income x Time |  |  | 0.04 *** |  |
|  |  |  | (0.03 – 0.04) |  |
| Education x Time |  |  |  | 0.04 *** |
|  |  |  |  | (0.04 – 0.05) |
| **Random Effects** |  |  |  |  |
| σ2 | 0.54 | 0.54 | 0.54 | 0.54 |
| τ00 | 0.21 county | 0.21 county | 0.21 county | 0.21 county |
|  | 0.06 state | 0.06 state | 0.06 state | 0.06 state |
| ICC | 0.33 | 0.33 | 0.33 | 0.33 |
| N | 2028 county | 2028 county | 2028 county | 2028 county |
|  | 51 state | 51 state | 51 state | 51 state |
| Observations | 89140 | 89140 | 89140 | 89140 |
| Marginal R2 / Conditional R2 | 0.330 / 0.552 | 0.331 / 0.553 | 0.331 / 0.554 | 0.331 / 0.554 |

*Note:* **p* < 0.05, ***p* < 0.01, ****p* < 0.001; 95% Confidence Intervals given in parentheses

Table H. Social Distancing (Reduction in Non-Essential Visitations) from April 13 to May 24 (Psychological & Cultural)

|  | **1** | **2** | **3** | **4** | **5** |
| --- | --- | --- | --- | --- | --- |
| **Predictors** | **β** | **β** | **β** | **β** | **β** |
| Intercept | -0.00 ** | -0.00 | -0.00 ** | -0.00 *** | -0.00 |
|  | (-0.06 – 0.05) | (-0.06 – 0.05) | (-0.06 – 0.05) | (-0.06 – 0.05) | (-0.06 – 0.05) |
| Time | -0.43 *** | -0.43 *** | -0.43 *** | -0.43 *** | -0.43 *** |
|  | (-0.43 – -0.42) | (-0.43 – -0.42) | (-0.43 – -0.42) | (-0.43 – -0.42) | (-0.43 – -0.42) |
| Shelter-in-Place Order | 0.02 | 0.02 | 0.02 | 0.02 | 0.02 |
|  | (-0.02 – 0.07) | (-0.02 – 0.07) | (-0.02 – 0.07) | (-0.02 – 0.07) | (-0.02 – 0.07) |
| Population Density | 0.00 | 0.00 | 0.00 | 0.00 | 0.00 |
|  | (-0.02 – 0.02) | (-0.02 – 0.02) | (-0.02 – 0.02) | (-0.02 – 0.02) | (-0.02 – 0.02) |
| County Population | 0.03 * | 0.03 * | 0.03 * | 0.03 * | 0.03 * |
|  | (0.01 – 0.05) | (0.01 – 0.05) | (0.01 – 0.05) | (0.01 – 0.05) | (0.01 – 0.05) |
| Elder (60+) Proportion | -0.13 *** | -0.13 *** | -0.13 *** | -0.13 *** | -0.13 *** |
|  | (-0.15 – -0.11) | (-0.16 – -0.11) | (-0.15 – -0.11) | (-0.15 – -0.11) | (-0.15 – -0.11) |
| Education Attainment | 0.23 *** | 0.23 *** | 0.23 *** | 0.23 *** | 0.23 *** |
|  | (0.20 – 0.26) | (0.20 – 0.26) | (0.20 – 0.26) | (0.20 – 0.26) | (0.20 – 0.26) |
| Income Inequality | 0.07 *** | 0.07 *** | 0.07 *** | 0.07 *** | 0.07 *** |
|  | (0.05 – 0.09) | (0.05 – 0.09) | (0.05 – 0.09) | (0.05 – 0.09) | (0.05 – 0.09) |
| Personal Income | -0.02 | -0.02 | -0.02 | -0.02 | -0.02 |
|  | (-0.05 – 0.01) | (-0.05 – 0.01) | (-0.05 – 0.01) | (-0.05 – 0.01) | (-0.05 – 0.01) |
| Unemployment | -0.03 * | -0.03 * | -0.03 * | -0.03 * | -0.03 * |
|  | (-0.06 – -0.00) | (-0.06 – -0.00) | (-0.06 – -0.00) | (-0.06 – -0.00) | (-0.06 – -0.00) |
| Democrat Proportion | 0.16 *** | 0.16 *** | 0.16 *** | 0.16 *** | 0.16 *** |
|  | (0.13 – 0.19) | (0.13 – 0.19) | (0.13 – 0.19) | (0.13 – 0.19) | (0.13 – 0.19) |
| Big 5 Neuroticism | 0.03 | 0.03 | 0.03 | 0.03 | 0.03 |
|  | (-0.02 – 0.08) | (-0.02 – 0.08) | (-0.02 – 0.08) | (-0.02 – 0.08) | (-0.02 – 0.08) |
| Tightness-Looseness | -0.10 *** | -0.10 *** | -0.10 *** | -0.10 * | -0.10 *** |
|  | (-0.16 – -0.05) | (-0.16 – -0.05) | (-0.16 – -0.05) | (-0.16 – -0.05) | (-0.16 – -0.05) |
| Collectivism | 0.07 * | 0.07 * | 0.07 * | 0.07 * | 0.07 |
|  | (0.01 – 0.12) | (0.01 – 0.12) | (0.01 – 0.12) | (0.01 – 0.12) | (0.01 – 0.12) |
| Democrat x Time |  | 0.08 *** |  |  |  |
|  |  | (0.07 – 0.08) |  |  |  |
| Neuroticism x Time |  |  | 0.00 |  |  |
|  |  |  | (-0.00 – 0.01) |  |  |
| Tightness x Time |  |  |  | -0.04 *** |  |
|  |  |  |  | (-0.05 – -0.04) |  |
| Collectivism x Time |  |  |  |  | 0.02 *** |
|  |  |  |  |  | (0.02 – 0.03) |
| **Random Effects** |  |  |  |  |  |
| σ2 | 0.54 | 0.54 | 0.54 | 0.54 | 0.54 |
| τ00 | 0.19 county | 0.19 county | 0.19 county | 0.19 county | 0.19 county |
|  | 0.03 state | 0.03 state | 0.03 state | 0.03 state | 0.03 state |
| ICC | 0.29 | 0.29 | 0.29 | 0.29 | 0.29 |
| N | 2019 county | 2019 county | 2019 county | 2019 county | 2019 county |
|  | 48 state | 48 state | 48 state | 48 state | 48 state |
| Observations | 88744 | 88744 | 88744 | 88744 | 88744 |
| Marginal R2 / Conditional R2 | 0.371 / 0.553 | 0.377 / 0.559 | 0.371 / 0.553 | 0.373 / 0.554 | 0.372 / 0.553 |

*Note:* **p* < 0.05, ***p* < 0.01, ****p* < 0.001; 95% Confidence Intervals given in parentheses

Table I. Social Distancing (Reduction in Non-Essential Visitations) from April 13 to May 24 (Health)

|  | **1** | **2** | **3** | **4** | **5** |
| --- | --- | --- | --- | --- | --- |
| **Predictors** | **β** | **β** | **β** | **β** | **β** |
| Intercept | 0.06 *** | 0.06 *** | 0.06 *** | 0.06 *** | 0.06 *** |
|  | (-0.01 – 0.12) | (-0.01 – 0.12) | (-0.01 – 0.12) | (-0.01 – 0.12) | (-0.01 – 0.12) |
| Time | -0.43 *** | -0.43 *** | -0.43 *** | -0.43 *** | -0.43 *** |
|  | (-0.43 – -0.42) | (-0.43 – -0.42) | (-0.43 – -0.42) | (-0.43 – -0.42) | (-0.43 – -0.42) |
| Shelter-in-Place Order | 0.04 | 0.04 | 0.04 | 0.04 | 0.04 |
|  | (-0.02 – 0.10) | (-0.02 – 0.10) | (-0.02 – 0.10) | (-0.02 – 0.10) | (-0.02 – 0.10) |
| Population Density | 0.01 | 0.01 | 0.01 | 0.01 | 0.01 |
|  | (-0.01 – 0.04) | (-0.01 – 0.04) | (-0.01 – 0.04) | (-0.01 – 0.04) | (-0.01 – 0.04) |
| County Population | 0.05 *** | 0.05 *** | 0.05 *** | 0.05 *** | 0.05 *** |
|  | (0.03 – 0.07) | (0.03 – 0.07) | (0.03 – 0.07) | (0.03 – 0.07) | (0.03 – 0.07) |
| Elder (60+) Proportion | -0.14 *** | -0.14 *** | -0.14 *** | -0.14 *** | -0.14 *** |
|  | (-0.16 – -0.12) | (-0.16 – -0.12) | (-0.16 – -0.12) | (-0.16 – -0.12) | (-0.16 – -0.12) |
| Education Attainment | 0.31 *** | 0.31 *** | 0.31 *** | 0.31 *** | 0.31 *** |
|  | (0.28 – 0.35) | (0.28 – 0.35) | (0.28 – 0.35) | (0.28 – 0.35) | (0.28 – 0.35) |
| Income Inequality | 0.07 *** | 0.07 *** | 0.07 *** | 0.07 *** | 0.07 *** |
|  | (0.05 – 0.10) | (0.05 – 0.10) | (0.05 – 0.10) | (0.05 – 0.10) | (0.05 – 0.10) |
| Personal Income | 0.01 | 0.01 | 0.01 | 0.01 | 0.01 |
|  | (-0.02 – 0.04) | (-0.02 – 0.03) | (-0.02 – 0.03) | (-0.02 – 0.03) | (-0.02 – 0.04) |
| Unemployment | -0.02 | -0.02 | -0.02 | -0.02 | -0.02 |
|  | (-0.05 – 0.01) | (-0.05 – 0.01) | (-0.05 – 0.01) | (-0.05 – 0.01) | (-0.05 – 0.01) |
| Preventable Hospitalization | -0.03 * | -0.03 | -0.03 * | -0.03 * | -0.03 * |
|  | (-0.05 – -0.00) | (-0.05 – -0.00) | (-0.05 – -0.00) | (-0.05 – -0.00) | (-0.05 – -0.00) |
| Vaccination | 0.08 *** | 0.08 *** | 0.08 * | 0.08 *** | 0.08 *** |
|  | (0.05 – 0.10) | (0.05 – 0.10) | (0.05 – 0.10) | (0.05 – 0.10) | (0.05 – 0.10) |
| Fair/Poor Health | 0.18 *** | 0.18 *** | 0.18 *** | 0.18 *** | 0.18 *** |
|  | (0.14 – 0.23) | (0.14 – 0.23) | (0.14 – 0.23) | (0.14 – 0.23) | (0.14 – 0.23) |
| At-Risk Preparedness Index | 0.08 ** | 0.08 ** | 0.08 ** | 0.08 *** | 0.08 ** |
|  | (0.03 – 0.12) | (0.03 – 0.12) | (0.03 – 0.12) | (0.03 – 0.12) | (0.03 – 0.12) |
| Surveillance & Epidemiological Index | -0.02 | -0.02 | -0.02 | -0.02 | -0.02 |
|  | (-0.08 – 0.05) | (-0.08 – 0.05) | (-0.08 – 0.05) | (-0.08 – 0.05) | (-0.08 – 0.05) |
| Hospitalization x Time |  | -0.01 *** |  |  |  |
|  |  | (-0.02 – -0.01) |  |  |  |
| Vaccination x Time |  |  | 0.03 *** |  |  |
|  |  |  | (0.02 – 0.03) |  |  |
| At-Risk x Time |  |  |  | 0.05 *** |  |
|  |  |  |  | (0.04 – 0.05) |  |
| Surveillance x Time |  |  |  |  | -0.00 |
|  |  |  |  |  | (-0.01 – 0.00) |
| **Random Effects** |  |  |  |  |  |
| σ2 | 0.54 | 0.54 | 0.54 | 0.54 | 0.54 |
| τ00 | 0.19 county | 0.19 county | 0.19 county | 0.19 county | 0.19 county |
|  | 0.06 state | 0.06 state | 0.06 state | 0.06 state | 0.06 state |
| ICC | 0.31 | 0.31 | 0.31 | 0.32 | 0.31 |
| N | 2025 county | 2025 county | 2025 county | 2025 county | 2025 county |
|  | 51 state | 51 state | 51 state | 51 state | 51 state |
| Observations | 89023 | 89023 | 89023 | 89023 | 89023 |
| Marginal R2 / Conditional R2 | 0.339 / 0.546 | 0.339 / 0.547 | 0.340 / 0.547 | 0.341 / 0.549 | 0.339 / 0.546 |

*Note:* **p* < 0.05, ***p* < 0.01, ****p* < 0.001; 95% Confidence Intervals given in parentheses
